# Integrated transcriptomic and metabolomic analysis of resistant and susceptible *Nicotiana tabacum* L. reveals the mechanisms of selenium-induced disease resistance to *Phytophthora nicotianae*

**DOI:** 10.3389/fpls.2025.1663346

**Published:** 2025-10-08

**Authors:** Kai Zhang, Xiaohan Ma, Jiaying Li, Fazhan Wang, Wenchao Wang, Jiashu Tian, Huanyu Teng, Yingjie Liu, Jiayang Xu, Huiwei Niu, Wei Jia

**Affiliations:** ^1^ College of Tobacco Science, Henan Agricultural University, Zhengzhou, Henan, China; ^2^ Staff Development Institute of China National Tobacco Corporation, Zhengzhou, Henan, China; ^3^ Henan Province Tobacco Company, Sanmenxia, Henan, China; ^4^ College of Resources and Environment, Henan Agricultural University, Zhengzhou, Henan, China

**Keywords:** selenium, tobacco black shank, disease resistance, transcriptome sequencing, metabolomics

## Abstract

Tobacco black shank, a destructive soil-borne disease caused by *Phytophthora nicotianae*, severely impacts tobacco production. Selenium (Se) is a beneficial trace element known to enhance plant stress resistance. While previous studies indicated Se’s efficacy against black shank, its differential effects on tobacco varieties with contrasting innate resistance remain unexplored. Herein, we integrated physiological, transcriptomic, and metabolomic analyses to elucidate the mechanisms of Se-induced resistance in a resistant (K326) and a susceptible (Zhongyan 100) tobacco cultivar. Our results showed that foliar application of 8 mg/L Se significantly reduced disease incidence and enhanced antioxidant enzyme activities, membrane stability, and accumulation of protective compounds in both cultivars. Multi-omics analyses revealed that Se potently enhanced resistance in K326 by synergistically upregulating the phenylpropanoid biosynthesis pathway, promoting the synthesis of lignin precursors and phenolic acids, and maintaining purine metabolism to ensure energy supply. In contrast, the susceptible cultivar Zhongyan 100 showed limited metabolic capacity to translate Se-induced transcriptional changes into effective defence metabolite accumulation. These findings provide novel insights into the genotype-dependent mechanisms of Se-induced resistance and highlight the potential of precision Se application as a strategy to bolster defence in resistant cultivars against soil-borne diseases.

## Graphical Abstract

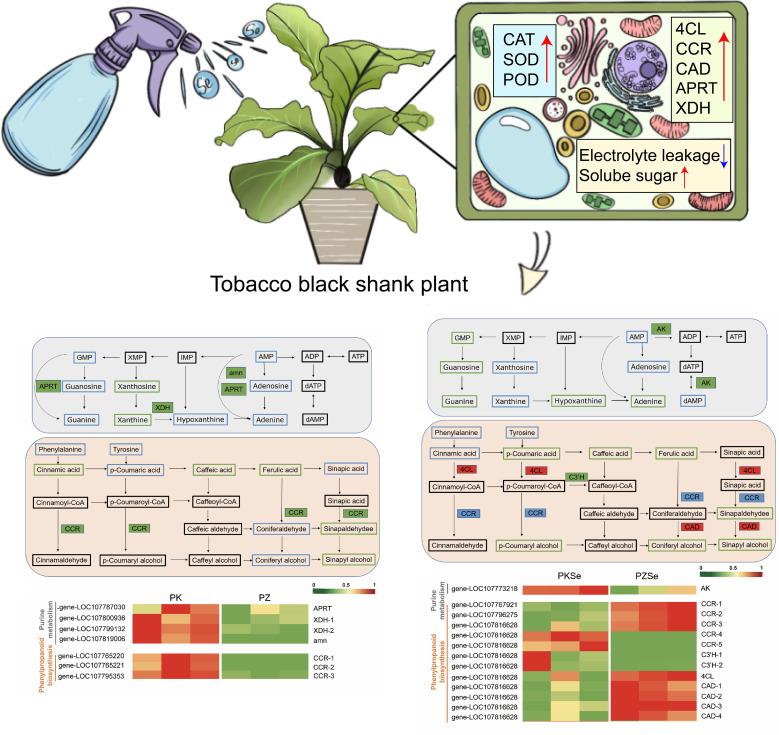

## 1 Introduction

Tobacco black shank, caused by *P. nicotianae*, is a serious soil-borne disease that can easily cause outbreaks in high-temperature and high-humidity weather (Wang et al., 2022). The oospores and chlamydospores produced by *P. nicotianae* usually accumulate within the root cap and wounds of the tobacco plant. Then, the produced mycelia enter cells, leading to plant dwarfing, yellowing of leaves, and necrosis of the stem base (Gallup et al., 2018; Kamoun, 2006). *P. nicotianae* can occur throughout the entire growth period of tobacco plants. Dissection of infected stems reveals a black-brown, dry stem pulp that is shrunken into a disc shape, with white mycelia between the discs (Liu et al., 2020). Owing to their strong viability and infectivity, oospores and chlamydospores can survive in adverse environments for several months (Gallup, 2009). Therefore, it is difficult to prevent and control tobacco black shank disease, which inevitably causes severe economic losses to the tobacco industry.

Currently, the prevention and control of tobacco black shank disease mainly involve cultivation measures, the selection and cultivation of disease-resistant varieties, and chemical and biological control. Although cultivation measures are useful in the management of black shank disease, they are unable to provide sustainable control of disease outbreaks (Gai et al., 2023). Chemical control has become the top choice for tobacco farmers to combat tobacco black shank because of its high efficiency and convenience. Nevertheless, the long-term use of fungicides not only causes serious ecological pollution but also increases the resistance of pathogenic bacteria (Kamoun et al., 2015; Qu et al., 2016). Recently, biological control has attracted attention because of its safety and low degree of pollution. However, biological control is still in an early stage of development and requires significant resources to achieve field application (Ma et al., 2023). Consequently, there is an urgent need to identify an efficient, eco-friendly, and cost-effective approach for preventing and controlling tobacco black shank. Harnessing host resistance has been demonstrated to be the most economical, effective, and environmentally friendly solution (Li et al., 2021; Piaskowska et al., 2021; Zhang et al., 2023).

Selenium (Se) is an essential trace element for life processes in both animals and plants (Chen et al., 2020). Plants absorb selenium from the soil by their roots, mainly in the forms of selenate, selenite, and a small quantity of organic selenium compounds (Peng et al., 2017). The importance of Se as a plant fertilizer has received increasing attention. Foliar spraying with an appropriate concentration of sodium selenate has been shown to increase the net photosynthetic rate and promote photosynthetic carbon assimilation in wheat, thereby increasing yield and biomass (Lara et al., 2019). Similarly, sugarcane supplemented with sodium selenate accumulates higher amounts of sugar and starch in the leaves (de Araujo et al., 2023). Se participates in plant antioxidant systems and redox regulation, and at appropriate concentrations, it enhances resilience to abiotic stresses, including heavy metal toxicity, drought, salinity, and temperature extremes at appropriate concentrations (Kamran et al., 2020; Pokhrel et al., 2020; Sita et al., 2023). Notably, Se nanoparticles exhibit larvicidal activity against *Spodoptera litura* (Arunthirumeni et al., 2022), and soil selenium application strengthens rapeseed resistance to *Sclerotinia sclerotiorum* (Xu et al., 2020). However, excessive selenium disrupts metabolism through membrane peroxidation, causing phytotoxicity symptoms such as leaf wilting and bud developmental delays (Cabral Gouveia et al., 2020), necessitating precise dosage control in agricultural applications.

Previous studies have shown that there was variation in resistance to black shank disease among tobacco varieties (Zhang et al., 2020). The application of metal elements to tobacco can improve its resistance to black shank disease (Yu et al., 2024). Although the role of Se in preventing and controlling plant diseases has been widely proved, it is still unknown whether Se exhibits differential effects on tobacco black shank between different tobacco varieties. Therefore, this study employed physiological, transcriptomic, and metabolomic analyses to explore the antifungal effect of Se on resistant and susceptible tobacco varieties. The results not only fill gaps in knowledge regarding the effect of Se on different disease-resistant tobacco varieties but also provide guidance for the application of Se in plant disease.

## 2 Materials and methods

### 2.1 Experimental design and treatments

Two experiments (Experiments I and II) were performed in this study:

Experiment I (Identification of Disease-Resistant Tobacco Varieties): Four kinds of flue-cured tobacco varieties (K326, Honghuadajinyuan, Zhongyan 100, and Changbohuang) were used in this experiment. Previously isolated P. nicotianae was cultured on oatmeal agar (OA) medium at 28 °C until use. Seeds were surface sterilised with 10% (v/v) H_2_O_2_ for 10 min, followed by thorough rinsing with sterile distilled water. The sterilised seeds were then placed on seedling sponges and transferred to a growth chamber maintained at 28 °C/18 °C (day/night) with a 14-h photoperiod and light intensity of 200 μmol m^-^² s^-^¹. When the seedlings reached the four-leaf stage, morphologically uniform healthy individuals were selected and transplanted into soil-filled pots. Seven days after transplantation, the pathogen was inoculated at the stem base by the attachment method. Sampling and biochemical determinations were conducted according to experimental protocols, with three biological replicates per treatment.

Experiment II: On the basis of the results from experiment I, the disease-resistant cultivar K326 and susceptible cultivar Zhongyan 100 were selected for subsequent analysis. When the seedlings reached the six-leaf phenological stage, foliar applications of sodium selenite (Na_2_SeO_3_, Sinopharm Group Chemical Reagent Co., LTD, ≥99.0% purity) solutions containing 0 (control), 2, 4, 6, 8, or 10 mg Se/L were administered. Each plant received 20 mL of treatment solution daily for seven consecutive days, corresponding to total selenium depositions of 0, 40, 80, 120, 160, and 200 mg Se/plant, respectively. The control groups were maintained with equivalent volumes of deionised water. Pathogen inoculation via stem insertion was performed following the seventh treatment. Sampling and biochemical determinations were conducted following the same method as in Experiment I, with three biological replicates per treatment group.

Two tobacco varieties (K326 and Zhongyan 100) treated with selenium solutions at concentrations of 0 mg/L (control) and 8 mg/L were subjected to transcriptomic and metabolomic profiling. For each variety, the samples were divided into two experimental groups with three biological replicates per group. The treatment groups were as follows: K326 plants receiving 0 mg/L and 8 mg/L selenium were labelled PK and PKSe, respectively, while Zhongyan 100 plants under equivalent treatments were designated PZ and PZSe. All samples were collected at 5 days post inoculation (dpi), immediately flash-frozen in liquid nitrogen, and stored at -80°C until RNA extraction and metabolite analysis.

### 2.2 Determination of disease incidence

For each treatment, 50 uniformly grown tobacco seedlings were selected for infection with *P. nicotianae*. The incidence of black shank disease was assessed after inoculation. Resistance was identified on the basis of the disease incidence, which was calculated using the following formula: Disease incidence (%) = (number of diseased plants/total number of plants) × 100%.

### 2.3 Determination of physiological and biochemical indexes

To determine the contents of total flavonoids and phenols, 1 g of tobacco leaf tissue was homogenised in 10 mL of 1% hydrochloric acid-methanol solution. The mixture was then centrifuged at 12,000 ×g for 20 min at 4°C. After centrifugation, the supernatant was collected, and its absorbance was measured at 280 nm and 325 nm, corresponding to the total phenolic and flavonoid contents, respectively. Three biological replicates were performed for all sampling and measurements. The MDA content was determined using the thiobarbituric acid method (Draper et al., 1993). The soluble protein content was quantified by the Coomassie blue staining method (Bradford, 1976), and the soluble sugar content was analysed by anthrone colorimetry (Yemm and Willis, 1954). The activities of catalase (CAT), peroxidase (POD) and superoxide dismutase (SOD) were analysed using commercial kits (Nanjing Jiancheng Bioengineering Institute, China) following manufacturer protocols. The contents of hydrogen peroxide (H_2_O_2_) and superoxide anion (O_2_^-^) were determined using commercial assay kits (Nanjing Jiancheng Bioengineering Institute, China). Leaf relative conductivity was determined using a conductivity meter (Leici DDS-11A, Shanghai, China). Photosynthetic parameters, including net photosynthetic rate (*Pn*), stomatal conductance (*Gs*), intercellular CO_2_ concentration (*Ci*), and transpiration rate (*Tr*), were measured between 9:00 and 11:00 AM using a Li-6000 portable photosynthesis system (LI-COR Biosciences, USA).

### 2.4 Trypan blue staining

To further validate the conclusions, *in vitro* experiments were performed. Foliar application was performed using a selenium solution at the optimised concentration. Uniformly sized tobacco leaves were then placed in plastic culture boxes lined with moistened gauze to maintain humidity. Fresh 4-mm-diameter mycelial plugs were aseptically inoculated onto identical positions of the leaves. Post inoculation, the samples were sealed with parafilm and incubated in a growth chamber at 28°C for 48 h. Leaf samples were stained with 0.05% (w/v) trypan blue solution (prepared in glycerol-lactic acid-phenol, 1:2:0.5 v/v) for 30 min, then destained in chloral hydrate solution (2.5 g/mL) for 48 hr to remove excess dye. The experimental procedure adhered to the methodology outlined in Li et al (Li et al., 2019). Triplicate biological replicates were maintained for each experimental condition.

### 2.5 Transcriptome sequencing and differentially expressed gene analysis

Three biological replicates per treatment (tobacco leaf samples) were collected for RNA extraction and transcriptome sequencing. A total of 3 μg of RNA per sample was used as input material for library preparation. RNA integrity was evaluated using the RNA Nano 6000 Assay Kit on a Bioanalyzer 2100 system (Agilent Technologies, Santa Clara, CA, USA). Sequencing libraries were constructed with the NEBNext Ultra RNA Library Prep Kit for Illumina (New England Biolabs, Ipswich, MA, USA) following experimental protocols outlined by the manufacturer, utilising uniquely assigned index codes to enable sample demultiplexing. Indexed libraries were clustered using the TruSeq PE Cluster Kit v3-cBot-HS on an Illumina cBot Cluster Generation System. After clustering, the libraries were sequenced on an Illumina NovaSeq 6000 platform to generate 150 bp paired-end reads. The raw sequencing data were processed to obtain clean reads by removing adapter-contaminated reads, poly-N sequences (>10% N content), and low-quality reads (Q score <20 for >50% bases). All RNA-seq data have been deposited in the NCBI SRA database under BioProject accession number PRJNA913648. Clean reads were aligned to the *Nicotiana tabacum* reference genome (TN90 assembly) using HISAT2, and gene expression levels were quantified as fragments per kilobase of per million mapped reads (FPKM). Differential expression analysis was conducted using DESeq2R (v1.16.1) in R. Gene Ontology (GO) enrichment was performed with clusterProfiler (v3.18.1) incorporating gene length bias correction. KEGG pathway enrichment was analysed using the same package with Fisher’s exact test.

To validate the reliability of the transcriptomic data, qRT–PCR was used to measure the expression levels of selected key regulatory genes. The relative expression trends between the transcriptomic samples and the qPCR data were highly consistent (**Supplementary Figure S1**), with Pearson correlation coefficients (R2) reaching 0.9781 and 0.9766 for the two experimental groups, respectively. These results statistically validated the technical reliability of the transcriptomic dataset.

### 2.6 Metabolome sequencing analysis

Three-gram aliquots from each treatment group were cryogenically pulverised in liquid nitrogen and extracted with 70% (v/v) methanol aqueous solution. The homogenate was centrifuged at 10,000 ×g for 15 min at 4°C. The resulting supernatant was collected, filtered and analysed using an LC–ESI–MS/MS system for tobacco stem tissue metabolite profiling. Quantitative analysis was performed in MRM mode with triple quadrupole mass spectrometry. Peak alignment and calibration were executed on the basis of retention time stability and chromatographic peak integrity. Differentially abundant metabolite screening was conducted through interquartile range normalisation across control groups. Principal component analysis (PCA) was then performed on all samples to assess the overall metabolic differences among the groups. Qualitative analysis of the primary and secondary mass spectrometry data was conducted using the in-house MWDB database (MetWare Biotechnology Co., Ltd., Wuhan, China) and publicly available metabolite databases.

### 2.7 Joint analysis of transcriptomics and metabolomics

Integrated multiomics analysis of differentially expressed genes (DEGs) and differentially abundant metabolites (DMs) was performed on the basis of the transcriptomic and metabolomic profiling results. The identified molecular components were mapped onto single or composite KEGG pathway diagrams, enabling systematic visualisation of upstream–downstream regulatory relationships between gene expression variations and metabolic alterations.

### 2.8 Fluorescence quantitative PCR validation analysis

To validate the transcriptomic results, quantitative real-time PCR (qRT–PCR) was used to measure the expression levels of selected key regulatory genes. Sample preparation for qRT–PCR was identical to that for RNA-seq. Total RNA was extracted from hyphal tissues using the Total RNA Isolation Kit QuantiFast^®^ TSYB^®^ Green PCR Kit (Qiagen, Germany) following the instructions provided with the RNA isolation kit. The ReverTra Ace qPCR RT Kit (Toyobo, Osaka, Japan) was subsequently used to reverse transcribe RNA into cDNA for further qRT–PCR analysis. SYBR qPCR Master Mix (Vazyme, Nanjing, China) was used for qRT–PCR detection. Specific primer sequences were designed using Primer Premier 5 (PREMIER Biosoft International, CA, USA), and all the primers used are listed in **Supplementary Table S1**. Each treatment sample included three biological replicates. Relative expression levels were calculated using the 2^−ΔΔCT^ method.

## 3 Results

### 3.1 Identification of resistance to *P. nicotianae* among different tobacco varieties

After inoculation with *P. nicotianae*, the disease incidence rates decreased in the following order: Zhongyan 100 > Changbohuang > Hongda > K326. The disease incidence rate of Zhongyan 100 reached 94.00%, followed by Changbohuang, whereas the lowest incidence rate of 74.00% was observed in K326. These results indicated that the K326 cultivar presented relatively strong disease resistance, whereas Zhongyan 100 presented the weakest resistance (**Supplementary Table S2**).

**Figure 1:**
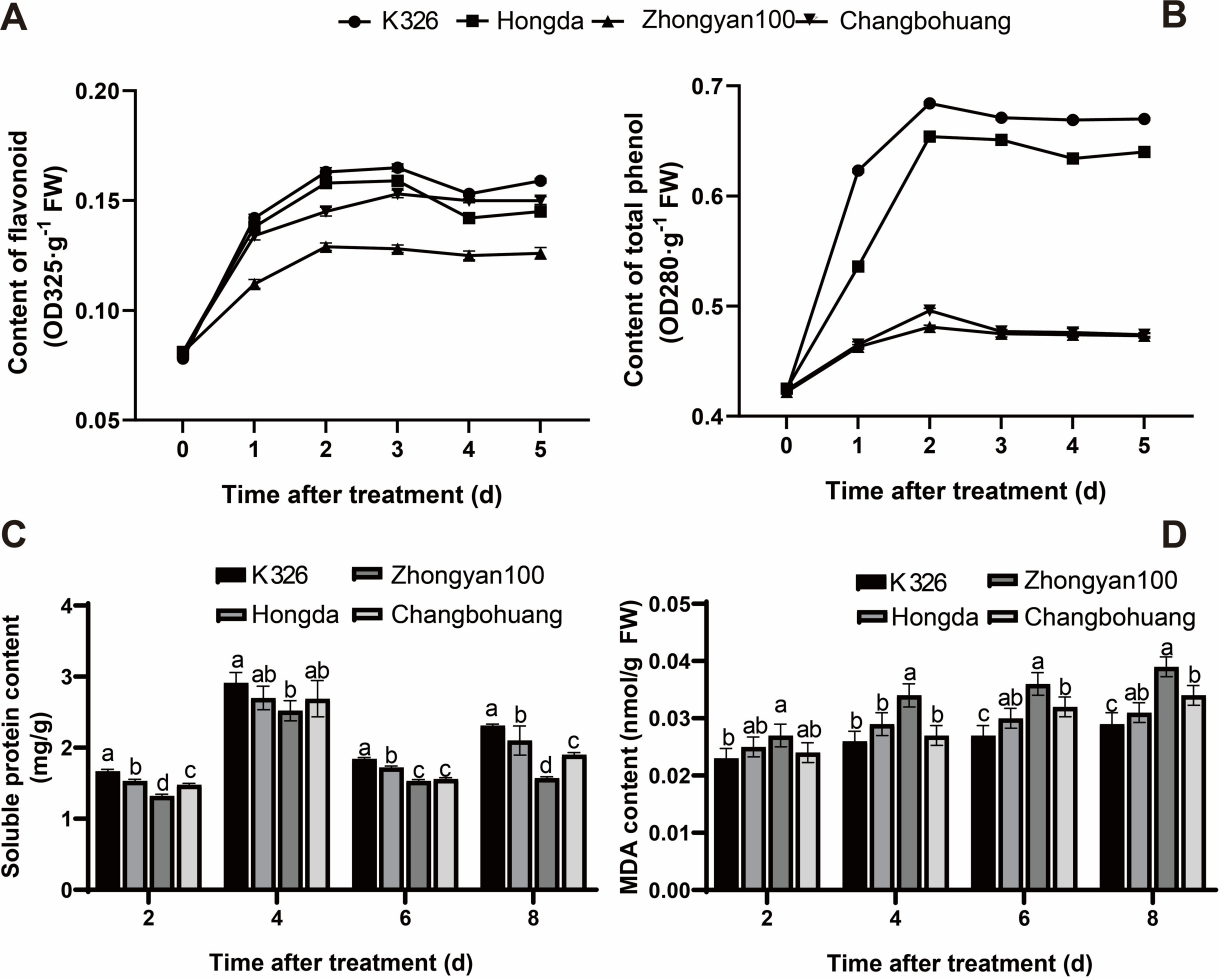
Effects of *P. nicotianae* infection on physiological indexes of different tobacco varieties. The physiological indexes assessed were flavonoid content **(A)**, total phenol content **(B)**, soluble protein content **(C)**, and MDA content **(D)**. Different lowercase letters indicate significant differences among treatments at *p* < 0.05.

Under pathogen inoculation, the contents of total phenols and flavonoids in the four varieties initially increased rapidly and then stabilised with prolonged infection time. Among them, the variation ranges of these indicators in K326 and Hongda were slightly greater than those in the other varieties, while Zhongyan 100 presented the smallest changes. At 5 days post inoculation, the contents of both compounds decreased in the following order: K326 > Hongda > Changbohuang > Zhongyan 100 (**Figures 1A, B**). The soluble protein content in the leaves of all four varieties first increased but then decreased with infection duration, peaking at 4 dpi. Significant differences in soluble protein content were observed only between K326 and Zhongyan 100. Across the four infection stages, K326 presented 26.52%, 15.48%, 20.26%, and 47.13% higher soluble protein contents than Zhongyan 100. Over time, K326 presented progressively increasing protein accumulation, whereas Zhongyan 100 presented minimal changes at 8 dpi. The soluble protein levels in Hongda and Changbohuang were between those in K326 and Zhongyan 100, with trends consistent with flavonoid and total phenol dynamics (**Figure 1C**). The malondialdehyde (MDA) content in K326, Hongda, Changbohuang, and Zhongyan 100 increased by 26.09%, 24.00%, 44.44%, and 41.67%, respectively, from 2 to 8 dpi. Among the four varieties, Zhongyan 100 presented the greatest increase. By 8 dpi, the MDA content in Zhongyan 100 was 1.34 times greater than that in K326, indicating that *P. nicotianae* infection caused more severe damage to Zhongyan 100 than to K326 (**Figure 1D**).

Taken together, these results demonstrated that resistance to black shank is not only reflected by a lower disease incidence but also by a milder disease severity and a more robust physiological response upon infection. The cultivar K326, which exhibited the lowest disease incidence, also maintained higher levels of protective compounds (flavonoids, phenols, soluble proteins) and lower membrane damage (MDA content) after pathogen challenge, indicating a comprehensive resistant phenotype. In contrast, Zhongyan 100 showed the highest susceptibility both in terms of infection rate and physiological deterioration. Therefore, K326 and Zhongyan 100 were selected as representative resistant and susceptible cultivars, respectively, for subsequent experiments.

### 3.2 Impact of Se on disease incidence and the antioxidant system in tobacco

On the basis of the experimental results, K326 and Zhongyan 100 were selected as resistant and susceptible varieties, respectively, for subsequent investigations. The disease incidence statistics for tobacco seedlings following foliar application of selenium at various concentrations are presented in **Table 1**. With increasing Se concentration, the disease incidence in K326 and Zhongyan 100 decreased, and the incidence in plants treated with 8 mg/L and 10 mg/L Se was lower in both varieties. Based on disease incidence data (**Table 1**), 8 mg/L Se was selected for transcriptomic and metabolomic profiling as it conferred the highest disease control efficacy in both cultivars (38.00% and 74.00% incidence in K326 and Zhongyan 100, respectively). Notably, at 10 mg/L Se, disease incidence slightly increased (K326: 44.00%; Zhongyan 100: 72.00%), suggesting potential phytotoxicity risks consistent with reports of selenium-induced metabolic disruption at excessive concentrations (Cabral Gouveia et al., 2020). To avoid confounding stress responses in omics analyses, the optimal 8 mg/L dose was prioritised.

**Table 1:** Disease incidence in two tobacco varieties treated with different Se concentrations.

Species	Se concentration (mg/L)	Incidence number	Total	Incidence rate
K326	0	38	50	76.00%
	2	36	50	72.00%
	4	34	50	68.00%
	6	30	50	60.00%
	8	19	50	38.00%
	10	22	50	44.00%
Zhongyan 100	0	49	50	98.00%
	2	48	50	96.00%
	4	45	50	90.00%
	6	40	50	80.00%
	8	37	50	74.00%
	10	36	50	72.00%

**Figure 2:**
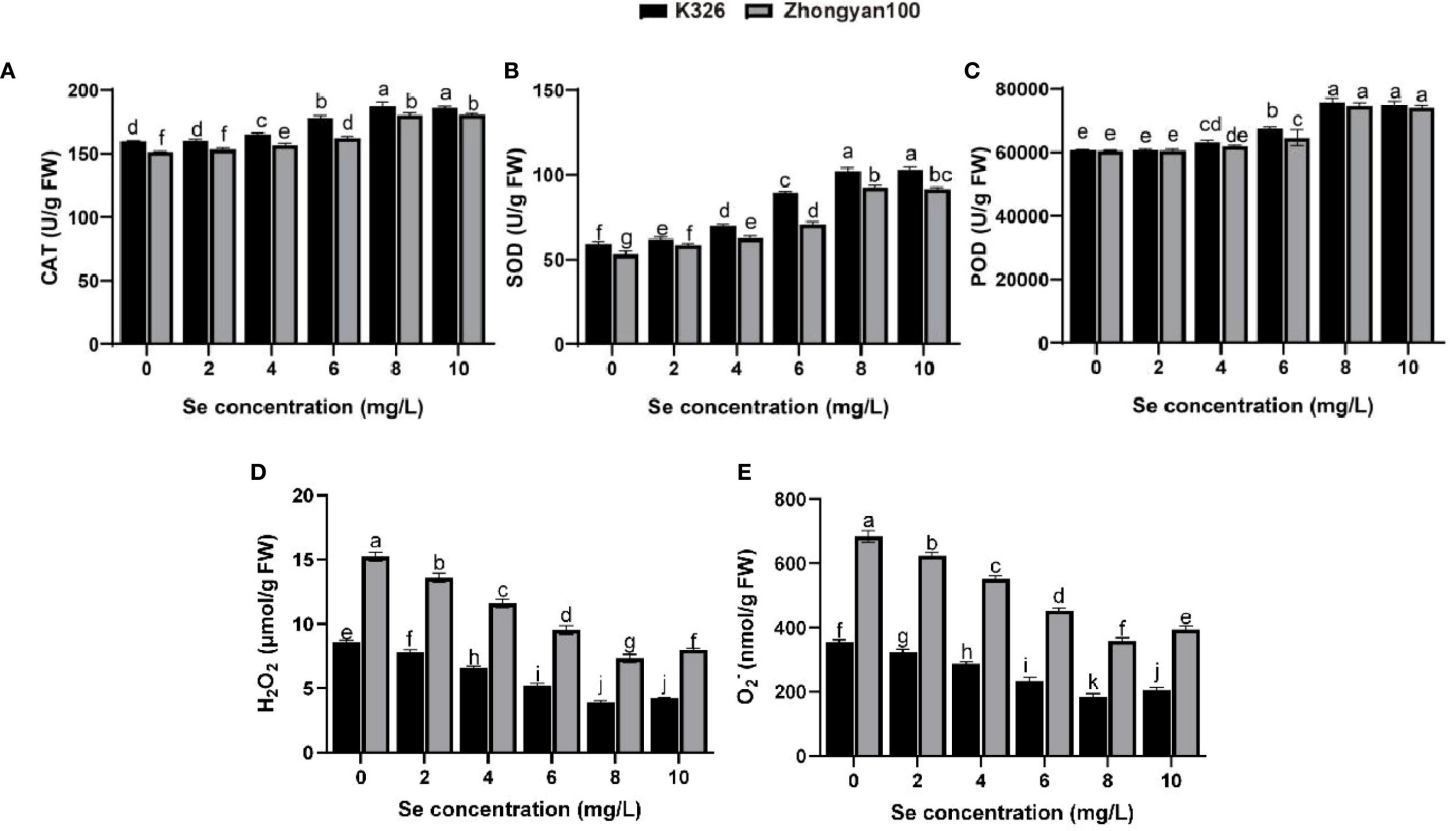
Effects of different selenium concentrations on antioxidant enzyme activities and ROS levels in tobacco varieties K326 and Zhongyan 100. **(A)** CAT activity; **(B)** SOD activity; **(C)** POD activity; **(D)** H_2_O_2_ content; **(E)** O_2_^-^ production rate. Different lowercase letters indicate significant differences among treatments at *p* < 0.05.

The activities of antioxidant enzymes (CAT, SOD, POD) and the accumulation of ROS in K326 and Zhongyan 100 under the six tested exogenous selenium concentrations are shown in **Figures 2A–E**. Antioxidant enzyme activity generally increased after selenium treatment, which was concomitant with a significant reduction in ROS levels (H_2_O_2_ and O_2_^-^). Compared with the control values, the SOD activity in both K326 and Zhongyan 100 plants significantly increased starting at 2 mg/L selenium, whereas the CAT and POD activities exhibited significant differences starting at 4 mg/L. Correspondingly, a significant decrease in H_2_O_2_ and O_2_^-^ content was observed at Se concentrations of 4 mg/L and above. At 8 mg/L selenium, the antioxidant enzyme activity in K326 increased by 3.94%, 10.45%, and 1.52% compared to those in Zhongyan 100, and the ROS content was 46.3% and 48.4% lower, respectively. Additionally, at 0 mg/L Se, the antioxidant enzyme activity of K326 was greater than that of Zhongyan 100, by 5.41%, 11.68%, and 0.28%, respectively; among these, the CAT and SOD activities differed significantly. This innate advantage was also reflected in the lower basal levels of ROS in the resistant K326 cultivar.

### 3.3 Effect of Se on the membrane system of tobacco leaves

**Figure 3:**
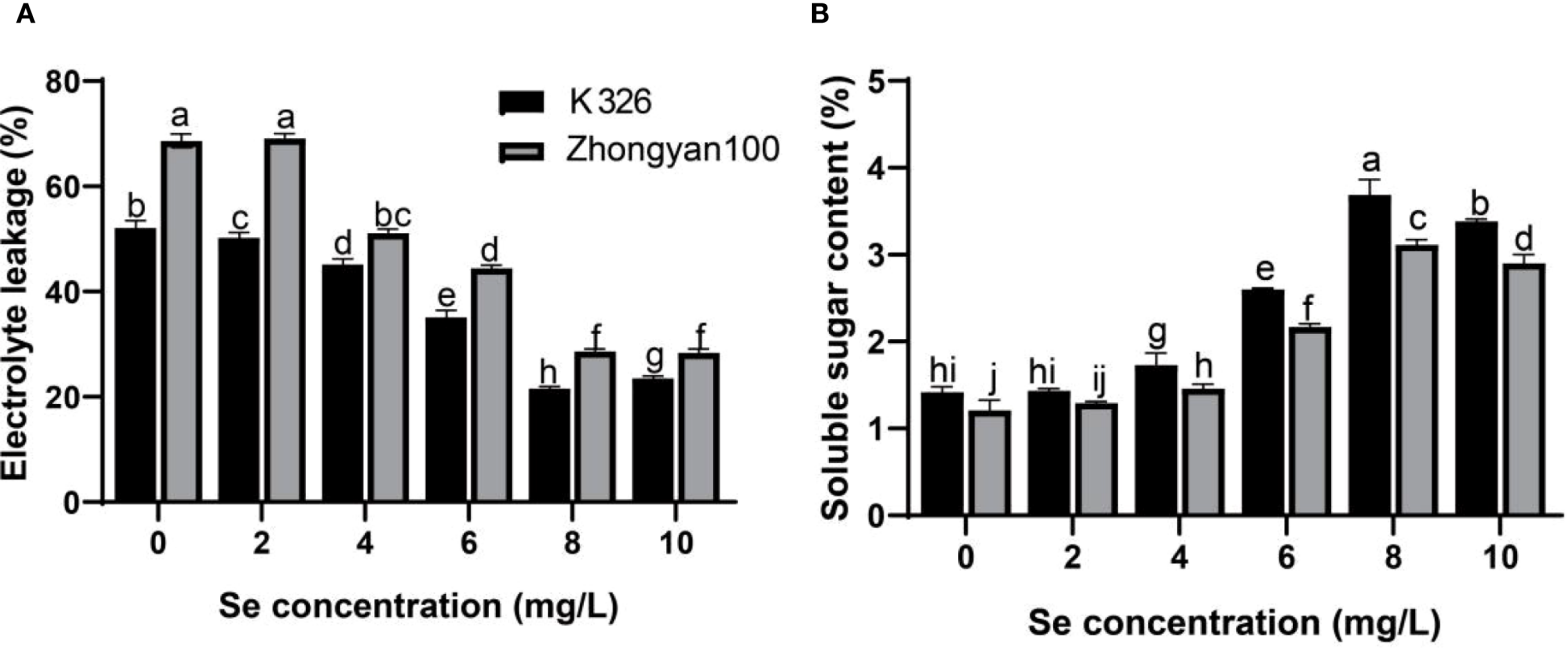
Effects of different Se concentrations on the electrical conductivity **(A)** and soluble sugar content **(B)** of the tobacco varieties K326 and Zhongyan 100. Different lowercase letters indicate significant differences among treatments at *p* < 0.05.

As shown in **Figures 3A, B**, in Zhongyan 100, selenium treatment (≥ 4 mg/L) significantly reduced the relative conductivity of tobacco leaves in Zhongyan 100 compared with the control. At selenium concentrations of 4, 6, 8, and 10 mg/L, the relative conductivity decreased by 34.29%, 54.52%, 139.71%, and 125.93%, respectively. K326 exhibited a similar trend, with the selenium treatments reducing the relative conductivity by 3.73%, 15.41%, 48.63%, 141.97%, and 128.47%, respectively, at the corresponding concentrations. The minimum relative conductivity and maximum reduction were observed in K326 in the 8 mg/L selenium treatment. Both cultivars presented increased soluble sugar contents following selenium treatment. In Zhongyan 100, the soluble protein content increased by 6.88%, 20.66%, 79.34%, 165.29%, and 166.12% across selenium concentrations, whereas K326 demonstrated increases of 0.94%, 21.83%, 83.33%, 159.86%, and 138.50%, respectively. Significant intervarietal differences (*p* < 0.05) were observed in both relative conductivity and soluble sugar content after treatment.

### 3.4 Effect of Se on photosynthetic characteristics of tobacco leaves under *P. nicotianae* infection

**Figure 4:**
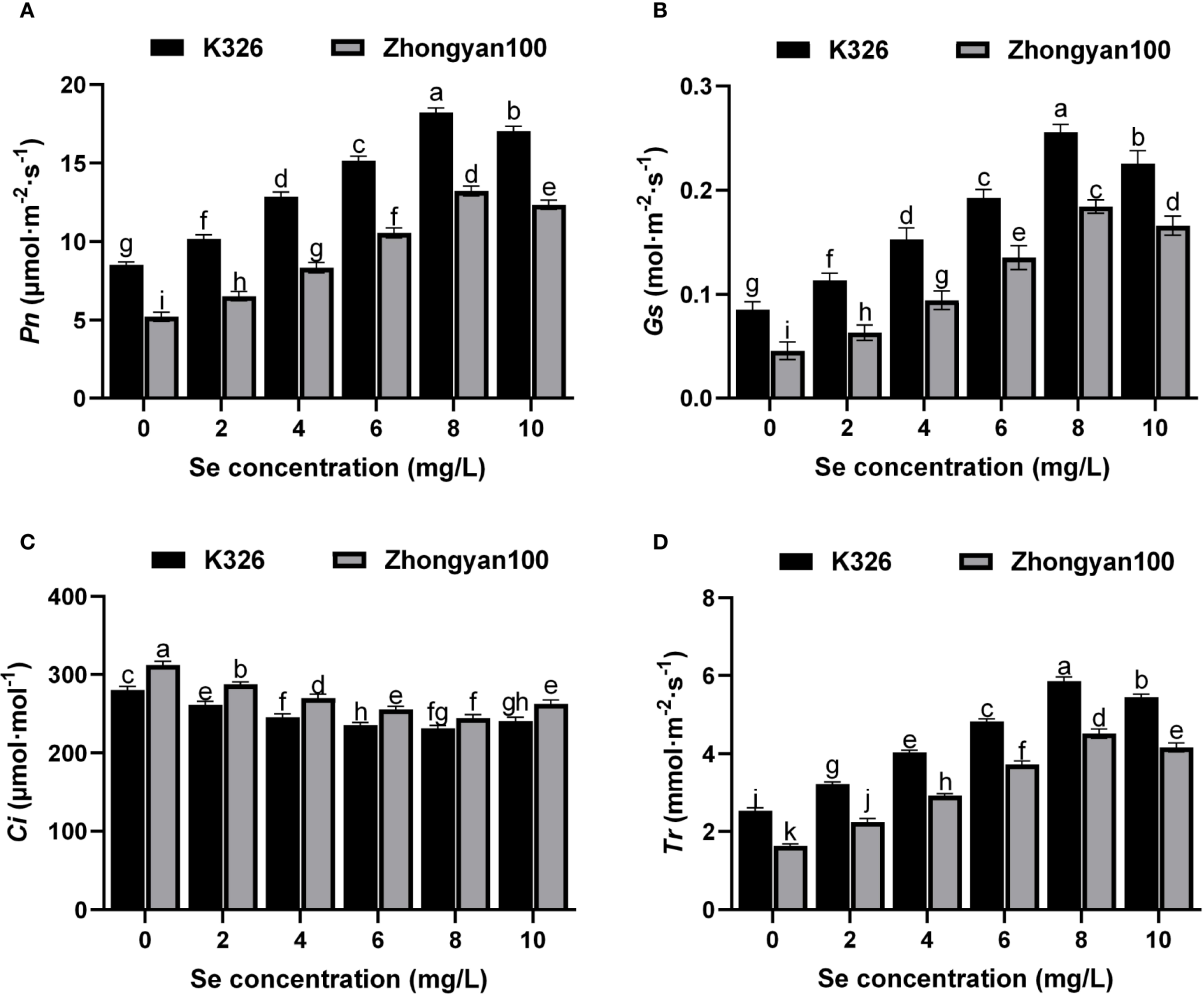
Effects of different selenium concentrations on photosynthetic parameters of resistant (K326) and susceptible (Zhongyan 100) tobacco varieties under *P. nicotianae* infection. **(A)** Net photosynthetic rate (*Pn*); **(B)** Stomatal conductance (*Gs*); **(C)** Intercellular CO_2_ concentration (*Ci*); **(D)** Transpiration rate (*Tr*). Different lowercase letters indicate significant differences among treatments at *p* < 0.05.

**Figures 4A–D** depict the photosynthetic parameters of resistant (K326) and susceptible (Zhongyan 100) tobacco cultivars under different exogenous selenium concentrations. Foliar application of selenium significantly alleviated the photosynthetic suppression induced by *P. nicotianae* infection in a concentration-dependent manner. The *Pn*, *Gs*, and *Tr* increased gradually with rising selenium concentrations from 0 to 8 mg/L, peaked at 8 mg/L, and slightly declined at 10 mg/L in both cultivars. In contrast, the *Ci* decreased continuously from 0 to 8 mg/L, reflecting enhanced mesophyll photosynthetic activity and CO_2_ utilisation efficiency, before a slight increase at 10 mg/L. Notably, the resistant cultivar K326 consistently exhibited superior photosynthetic performance over Zhongyan 100 across all selenium levels. At the optimal concentration of 8 mg/L Se, K326 showed significantly higher values in *Pn*, *Gs*, and *Tr* than Zhongyan 100, with increases of 37.8%, 39.1%, and 29.6% respectively. Correspondingly, *Ci* in K326 was 5.3% lower than in Zhongyan 100, further supporting its enhanced non-stomatal photosynthetic capacity. These results demonstrate that selenium application effectively mitigates pathogen-induced photosynthetic impairment by improving stomatal opening and reinforcing biochemical processes in mesophyll cells. The genotype-dependent response underscores the inherent advantage of K326 in utilising selenium to maintain robust photosynthetic performance under pathogen stress.

### 3.5 Effect of Se on the activity of tobacco cells

**Figure 5:**
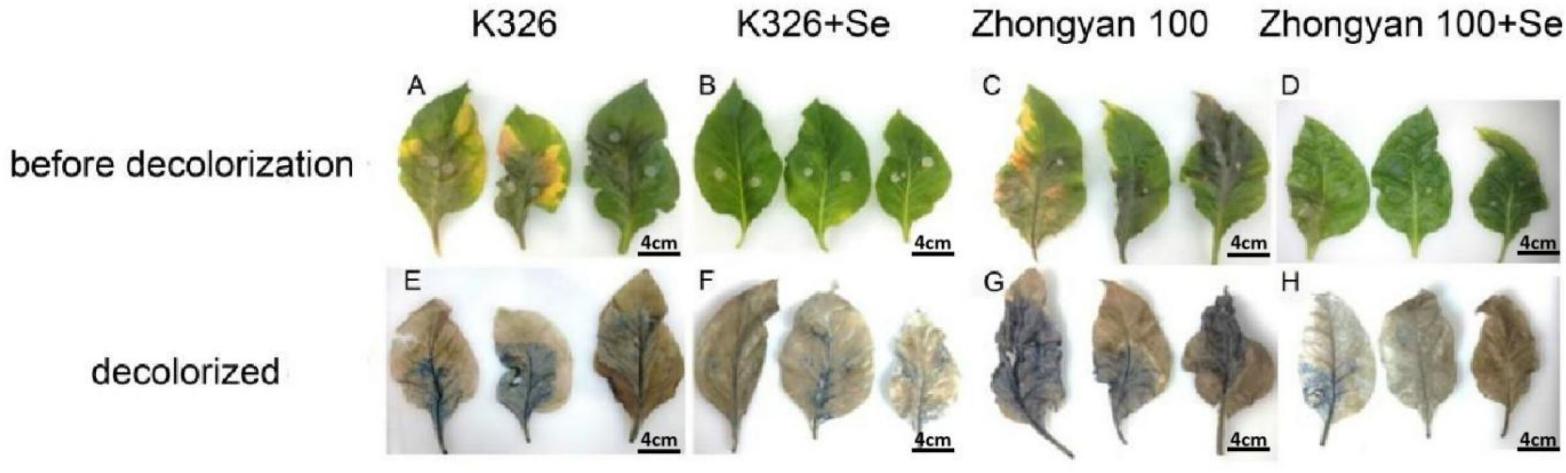
Trypan blue staining of two varieties of tobacco leaves under different treatments. **(A–D)** show K326, K326+Se, Zhongyan 100 and Zhongyan 100+Se before decolourisation, respectively; **(E–H)** show decolourised K326, K326+Se, Zhongyan 100 and Zhongyan 100+Se. Scale bars = 4 cm.

On the basis of the aforementioned experiments, tobacco leaves subjected to 8 mg/L selenium treatment were stained with trypan blue. The results demonstrated that prior to decolourisation, the leaves of both the K326 and Zhongyan 100 cultivars in the control group presented varying degrees of lesions caused by *P. nicotianae* infection. Following foliar application of 8 mg/L selenium solution, a significant reduction in lesion severity was observed (**Figures 5A–D**). The extent of lesions after decolourisation was represented by the blue attached area, and the overall severity of lesions decreased in the following order: Zhongyan 100 > K326 > Zhongyan 100+Se > K326+Se (**Figures 5E–H**). These findings indicated that selenium treatment substantially inhibited pathogen colonisation in both cultivars, thereby increasing the resistance of tobacco leaves to black shank disease. Notably, the improvement in the K326 cultivar was more pronounced than that in the Zhongyan 100 cultivar under selenium treatment.

### 3.6 Transcriptomic analysis

For transcriptomic analysis, total RNA was extracted from K326 and Zhongyan 100 tobacco varieties treated with 0 or 8 mg/L selenium combined with *P. nicotianae* infection for sequencing. The results demonstrated that clean reads accounted for more than 92.00% of the raw reads across all four treatment groups, yielding a total of 77.32 Gb of data. Each sample generated more than 6 Gb of clean reads, with Q20 scores surpassing 97.79% and Q30 scores above 93.50%, meeting the quality standards for transcriptome analysis (**Supplementary Table S3**). Furthermore, more than 94% of the clean reads were mapped to the reference genome in all samples. The uniform alignment rates across samples confirmed data comparability between the experimental groups (**Supplementary Table S4**).

**Figure 6:**
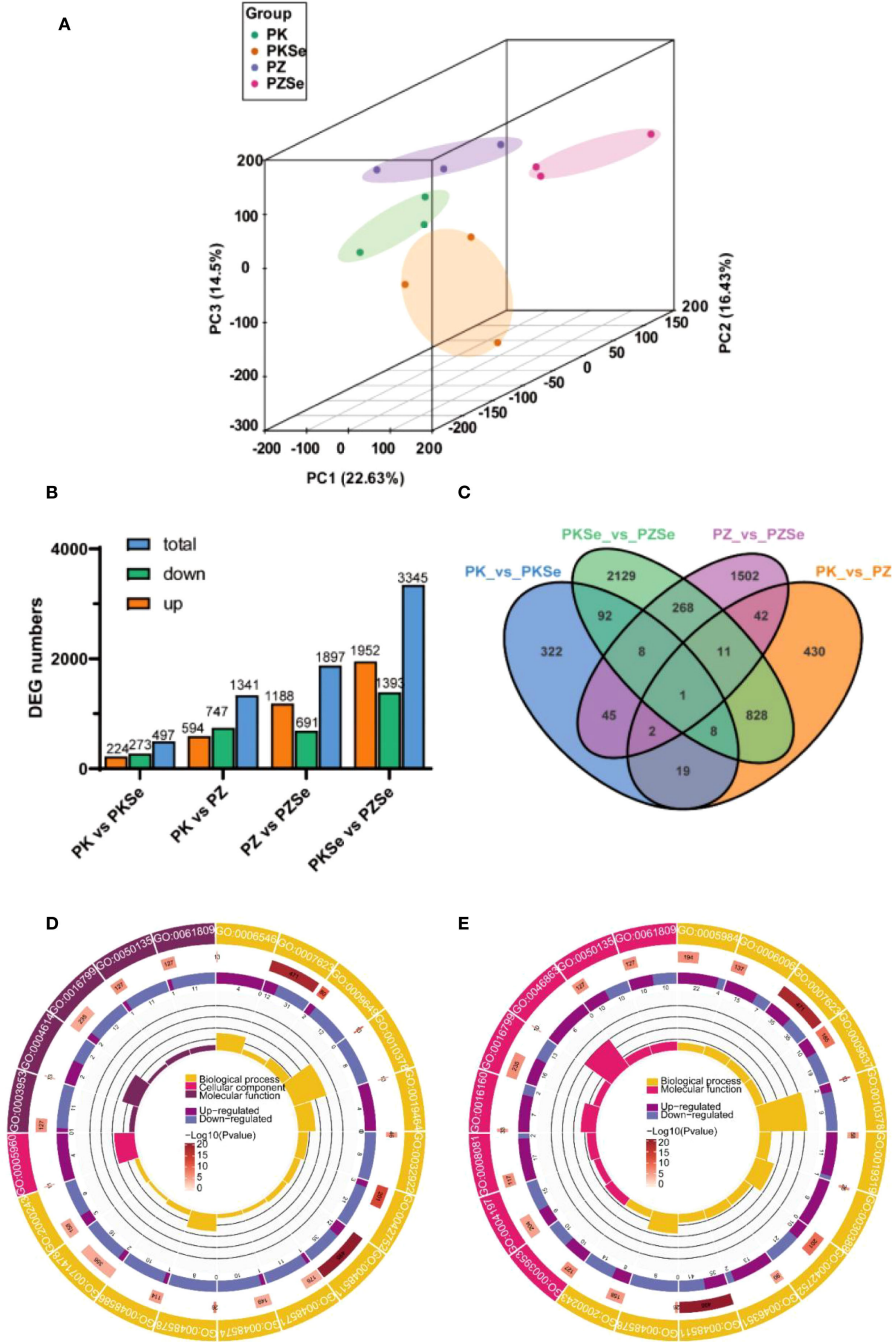
PCA and DEG quantity under different treatments. **(A)** PCA chart; **(B)** Upregulated, downregulated and total DEGs between different groups; **(C)** Venn diagram of DEGs between different groups. **(D)** GO functional classification diagram of DEGs in the PK vs. PZ groups. **(E)** GO functional classification diagram of DEGs in the PKSe vs. PZSe groups.

Principal component analysis (PCA) revealed distinct separations of the four treatment groups along PC1, PC2 and PC3 (**Figure 6A**). The quantitative distribution of DEGs, screened using defined parameters, is presented in **Figure 6B**. Subsequent analyses were conducted on these identified DEGs. Comparisons between varieties revealed 1,341 DEGs between PK and PZ (594 significantly upregulated, 747 downregulated) and 3,345 DEGs between PKSe and PZSe (1,952 upregulated, 1,393 downregulated). Intravariety selenium treatment comparisons revealed 497 DEGs between PK and PKSe (224 upregulated, 273 downregulated) and 1,897 DEGs between PZ and PZSe (1,188 upregulated, 691 downregulated). Venn diagram analysis (**Figure 6C**) revealed that PKSe vs. PZSe presented the greatest number of unique DEGs (1,027), whereas PK vs. PZ presented fewer unique DEGs (218), indicating substantial transcriptional reprogramming induced by selenium application in both tobacco cultivars.

Comparative analysis within each cultivar revealed that selenium application induced substantial transcriptional reprogramming. In the resistant cultivar K326, comparison between PKSe and PK groups identified 497 differentially expressed genes (DEGs), with 224 upregulated and 273 downregulated. In contrast, a more pronounced response was observed in the susceptible cultivar Zhongyan 100, where the PZSe vs PZ comparison yielded 1,897 DEGs (1,188 upregulated and 691 downregulated). This indicates a more extensive transcriptome-wide response to selenium in the susceptible variety under pathogen stress.

### 3.7 GO enrichment and functional annotation classification

In this study, the GOseq R package was used to perform functional annotation and classification of the identified DEGs. The results demonstrated that under *P. nicotianae* infection, 2500 Gene Ontology (GO) terms were enriched in the PZ group compared with the PK group, with 20 significantly enriched terms primarily clustered in biological processes and molecular functions. Comparative analysis of GO functional categories revealed that biological process terms were enriched in circadian rhythm (GO:0007623), rhythmic process (GO:0048511), glycine catabolism (GO:0006546), and temperature compensation of the circadian clock (GO:0010378). Upregulated genes dominated glycine catabolism, whereas other processes were predominantly downregulated; cellular component terms were enriched in glycine cleavage complex (GO:0005960), with exclusive upregulation; and molecular function terms were enriched in phosphoglucomutase activity (GO:0004614), NAD+ nucleosidase activity (GO:0003953), and NAD(P)+ nucleosidase activity (GO:0050135), which were predominantly associated with downregulated genes (**Figure 6D**).

Comparative analysis of the PKSe and PZSe groups revealed 3,560 enriched GO terms from DEGs, with 20 significantly enriched terms exclusively related to biological processes and cellular components. Biological process terms were predominantly associated with circadian rhythm (GO:0007623), rhythmic process (GO:0048511), temperature-compensated circadian clock (GO:0010378), positive regulation of long-day photoperiodism/flowering (GO:0048578), and fructose-1,6-bisphosphate metabolic process (GO:0030388), with predominant downregulation. Cellular component terms included hydrolase activity hydrolysing N-glycosyl compounds (GO:0016799), ribulose-1,5-bisphosphate carboxylase (GO:0046863), amylase activity (GO:0016160), and phosphodiester hydrolase activity (GO:0008081), which were primarily upregulated (**Figure 6E**). This pattern indicated conserved selenium-independent differential regulation of circadian/rhythmic processes across cultivars. Specifically, PK vs. PZ comparisons revealed glycinergic metabolic divergence with downregulation of NAD+ nucleotidase activity, whereas PKSe vs. PZSe comparisons demonstrated pronounced carbohydrate metabolic reprogramming, particularly in sugar metabolism pathways.

### 3.8 KOG annotation classification and KEGG enrichment analysis

**Figure 7:**
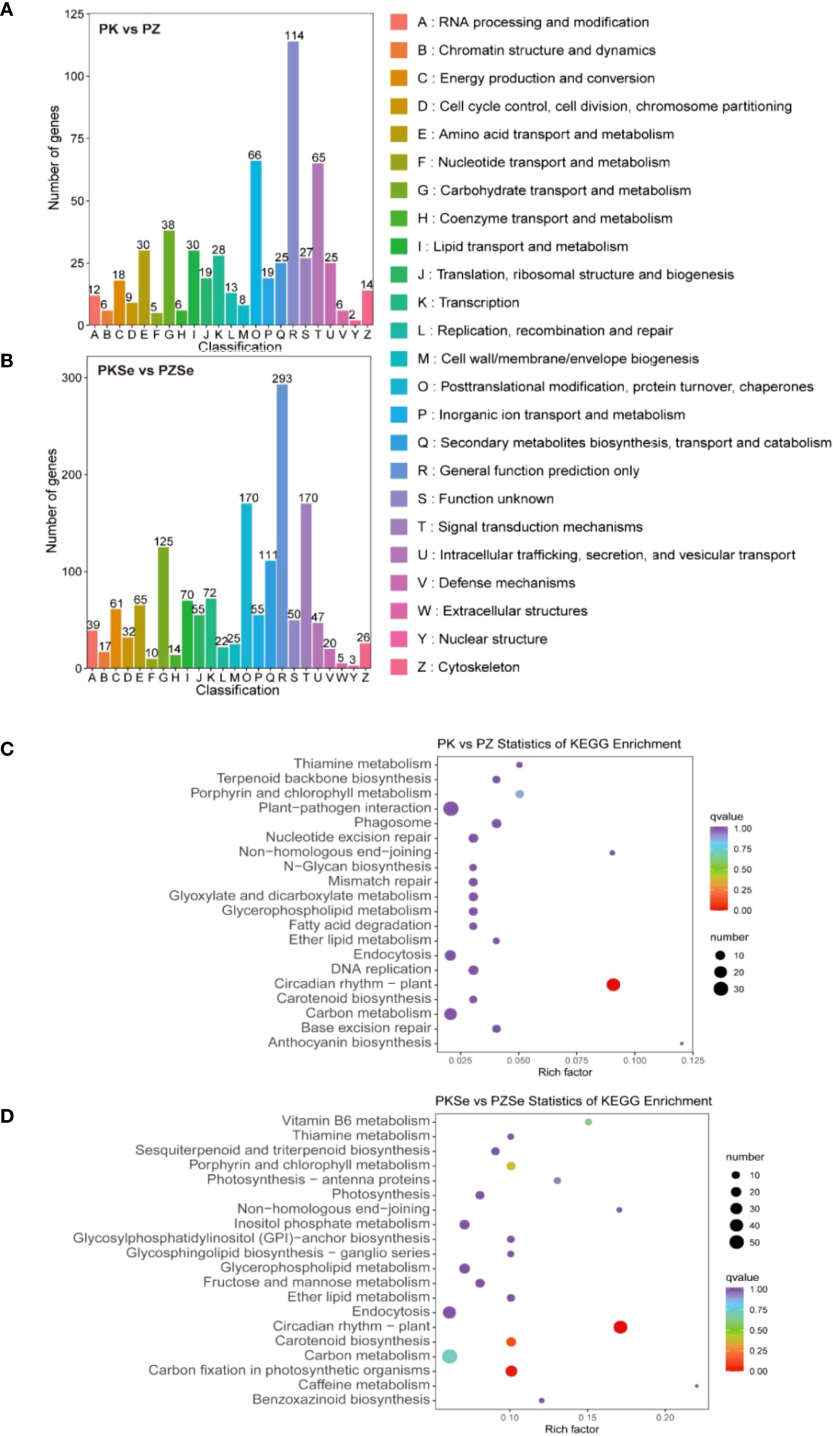
**(A)** KOG classification of DEGs in the PK vs. PZ groups. **(B)** KOG classification of DEGs in the PKSe vs. PZSe groups. **(C)** Statistical scatter plot of the KEGG pathways of DEGs in the PK vs. PZ groups. **(D)** Statistical scatter plot of the KEGG pathways of DEGs in the PKSe vs. PZSe groups.

A systematic comparison of DEGs across four experimental treatments against the KOG database revealed distinct functional annotations and categorical distributions. The PK vs. PZ and PKSe vs. PZSe comparisons were mapped to 23 and 24 KOG categories, respectively, with both groups exhibiting the highest gene counts in “General function prediction only” (114 vs. 293 genes). Shared enrichment was observed in three core functional categories: posttranslational modification, protein turnover, chaperones (66 vs. 170 genes), signal transduction mechanisms (65 vs. 170 genes), and carbohydrate transport and metabolism (38 vs. 125 genes). Notably, PK vs. PZ presented unique enrichment in amino acid transport and metabolism and lipid transport and metabolism, whereas PKSe vs. PZSe presented predominant annotations in secondary metabolite biosynthesis, transport and catabolism, and transcription-related functions, highlighting treatment-specific metabolic and regulatory divergence (**Figures 7A, B**).

To analyse the signalling pathways associated with the Se-induced DEGs in the two tobacco varieties, KEGG pathway annotation was performed. The results demonstrated that DEGs in the PK vs. PZ comparison were predominantly annotated to pathways including circadian rhythm in plants, plant-pathogen interaction, porphyrin and chlorophyll metabolism, phagosome, and base excision repair (**Figure 6C**). In contrast, DEGs in the PKSe vs. PZSe comparison were significantly enriched in circadian rhythm in plants, carbon fixation in photosynthetic organisms, vitamin B6 metabolism, carotenoid biosynthesis, and porphyrin and chlorophyll metabolism (**Figure 7D**). These findings indicate that the two tobacco genotypes exhibit divergent physiological and biochemical responses to *P. nicotianae* infection, particularly with respect to DNA damage repair mechanisms and pathogen defence signalling. Furthermore, exogenous selenium application induced genotype-specific alterations in the photosynthetic system and photosynthetic pigment synthesis pathways, underscoring the regulatory role of selenium in increasing photoprotective capacity and metabolic resilience under pathogen-induced stress.

KOG functional classification of the DEGs from intravariety comparisons (PKSe vs PK and PZSe vs PZ) further elucidated the impact of selenium. In K326 (PKSe vs PK), DEGs were prominently annotated to categories such as Posttranslational modification, protein turnover, chaperones and Signal transduction mechanisms. In Zhongyan 100 (PZSe vs PZ), besides these categories, a significant number of DEGs were also classified into Carbohydrate transport and metabolism and Secondary metabolites biosynthesis, transport and catabolism. This suggests that selenium influenced a broader spectrum of metabolic processes, including primary and secondary metabolism, in the susceptible cultivar.

### 3.9 Metabolomics analysis

**Figure 8:**
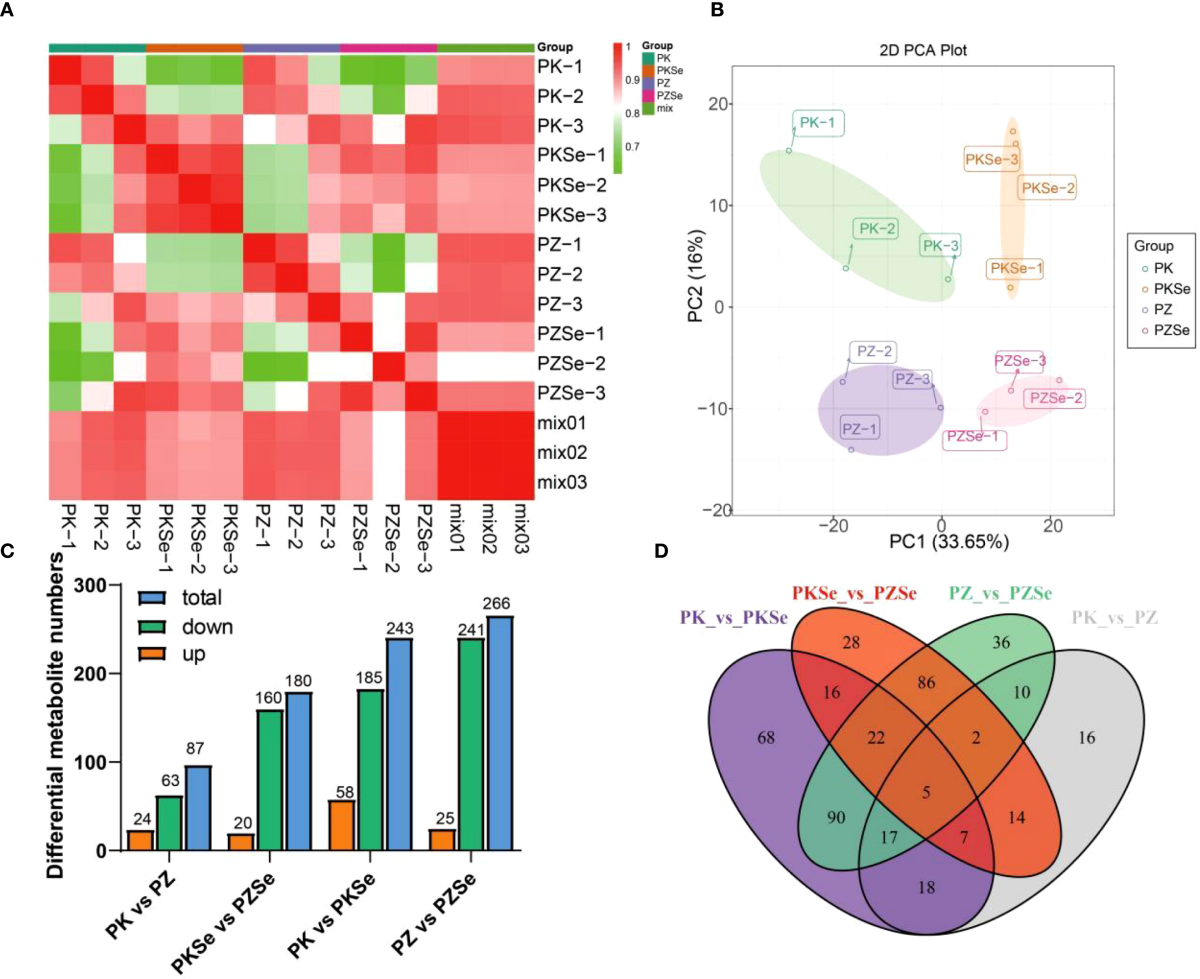
Statistics of the differential accumulation of metabolites. **(A)** Correlations between the three treatments and metabolites. **(B)** PCA of metabolomic data from the different treatments. **(C)** Statistical analysis of up- and downregulated differentially accumulated metabolites in the four treatment groups. **(D)** Venn diagram showing the differences between the four different groups.

To investigate metabolic alterations in the two tobacco varieties under *P. nicotianae* infection and selenium treatment, LC/MS-based broad-target metabolomics profiling was conducted across four experimental groups (PK, PZ, PKSe, and PZSe). Intragroup reproducibility and intergroup variability were assessed using Pearson correlation coefficients calculated via the cor function in R, revealing strong correlations among biological replicates within groups and significant metabolic divergence between varieties (**Figure 8A**). PCA further demonstrated clear separation among groups within a 95% confidence interval (**Figure 8B**), confirming the robustness and reproducibility of the dataset. Differentially abundant metabolite screening (thresholds: *p* < 0.05, |log2FC|≥ 1) revealed 87 differentially accumulated metabolites (DAMs) in PK vs. PZ (24 upregulated, 63 downregulated), 180 DAMs in PKSe vs. PZSe (20 upregulated, 160 downregulated), 241 DAMs in PK vs. PKSe (58 upregulated, 183 downregulated), and 266 DAMs in PZ vs. PZSe (25 upregulated, 241 downregulated) (**Figure 8C, D**). Venn diagram analysis revealed that, compared with pathogen inoculation alone, exogenous selenium application significantly increased the number of DAMs in both tobacco varieties, demonstrating the profound regulatory impact of selenium on intracellular metabolite homeostasis.

### 3.10 Classification and KEGG enrichment of metabolites

**Figure 9:**
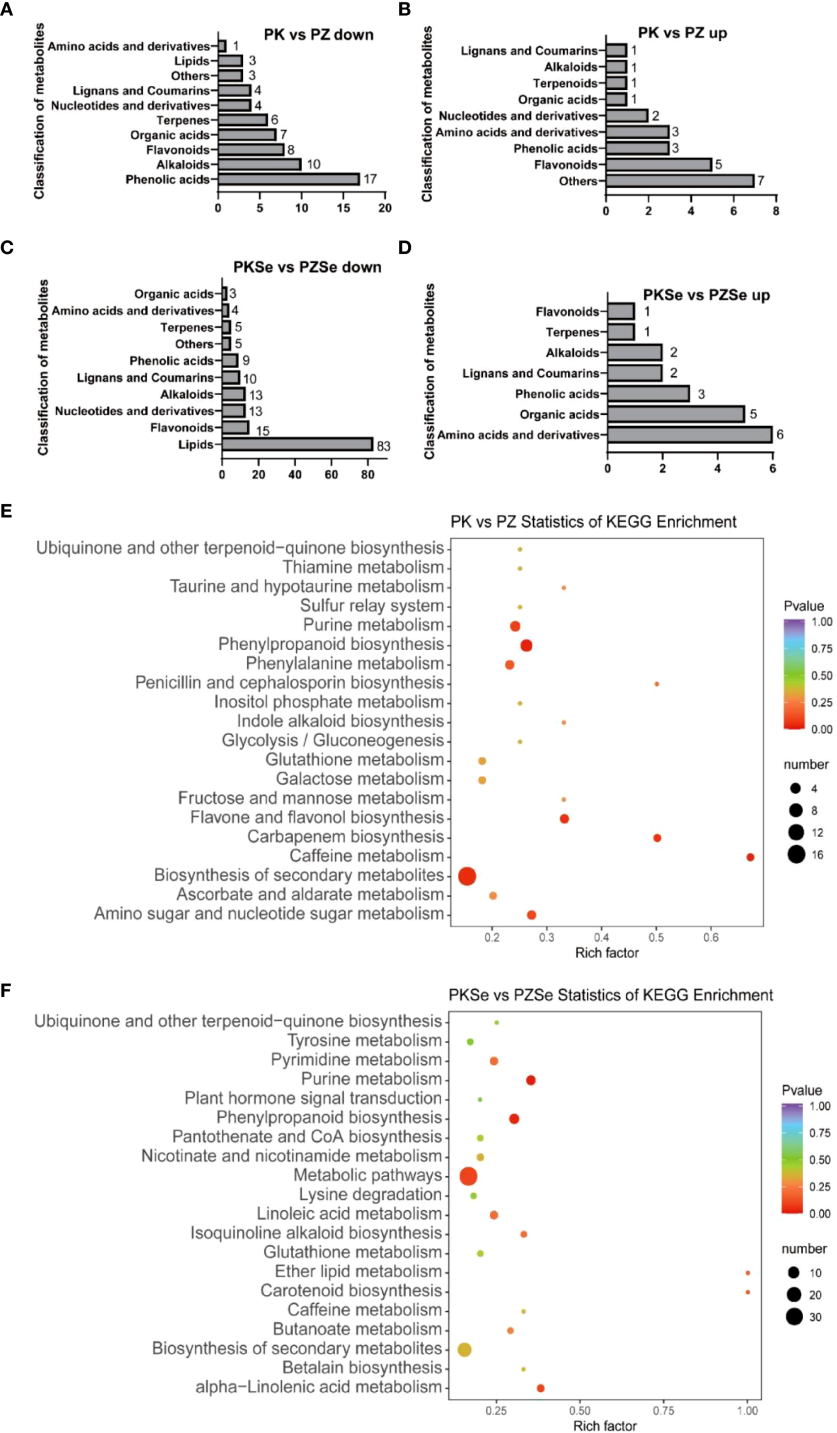
Quantitative statistics of the primary classification of differentially accumulated metabolites and KEGG enrichment pathways of DMs. **(A)** Metabolites downregulated in the PK vs. PZ group; **(B)** Metabolites upregulated in the PK vs. PZ group; **(C)** Metabolites downregulated in the PKSe vs. PZSe group; **(D)** Metabolites upregulated in the PKSe vs. PZSe group. **(E, F)** KEGG enrichment pathways of PK vs. PZ and PKSe vs. PZSe for the two groups of DMs.

As shown in **Figures 9A–D**, compared with the PK group, the PZ group presented 63 downregulated DMs annotated into 10 primary categories, predominantly phenolic acids (17 compounds), alkaloids (10), flavonoids (8), organic acids (7), and terpenes (6) (**Supplementary Table S5**). The top five downregulated metabolites by fold change were caffeoylcholine (alkaloid class), 6,7-dihydroxy-4-methylcoumarin (coumarin class), sinapyl alcohol (phenolic acid class), isorhamnetin-7-O-glucoside (flavonoid class), and hydroxyacetophenone (phenolic acid class). Conversely, 24 upregulated metabolites in the PZ vs. PK comparison were classified into 9 categories, primarily miscellaneous (7 compounds) and flavonoids (5), with the most significantly upregulated being L-tryptophan-O-diglucoside (amino acid and derivatives), L-proline (amino acid and derivatives), D-arabinose (miscellaneous), D-mannitol (miscellaneous), and D-fructose (miscellaneous) (**Supplementary Table S6**). In the PKSe vs. PZSe comparison, 160 downregulated metabolites were mapped to 10 primary categories, dominated by lipid (83 compounds), flavonoid (15), alkaloid (13), nucleotide and derivatives (13), lignin and coumarin (10), and phenolic acid classes (9). The six most downregulated metabolites included caffeoylcholine (alkaloid class), sinapyl alcohol (phenolic acid class), 6,7-dihydroxy-4-methylcoumarin (lignin and coumarin class), kaempferol-3-O-neohesperidoside-7-O-glucoside (flavonoid class), p-coumaric acid (phenolic acid class), and p-coumaroyl alcohol (phenolic acid class) (**Supplementary Table S7**). Among the 20 upregulated metabolites in this group, amino acid derivatives (6 compounds) and organic acids (5) were predominant, with the greatest accumulation observed in N-acetyl-L-leucine (amino acid derivatives), 5-O-ferulic acid (phenolic acid class), abscisic acid (organic acid class), resveratrol (alkaloid class), and scopolamine lactone 7-O-glucuronide (lignan and coumarin class) (**Supplementary Table S8**).

In the KEGG pathway enrichment analysis, DMs in the PK vs. PZ comparison were enriched primarily in phenylpropanoid biosynthesis, caffeine metabolism, carbapenem biosynthesis, biosynthesis of flavonoids and flavonols, purine metabolism, amino sugar and nucleotide sugar metabolism, and phenylalanine metabolism (**Figure 9E**). In contrast, DMs in the PKSe vs. PZSe comparison were significantly enriched in purine metabolism, phenylpropanoid biosynthesis, α-linolenic acid metabolism, ether lipid metabolism, carotenoid biosynthesis, and isoquinoline alkaloid biosynthesis (**Figure 9F**).

The impact of selenium on the metabolic profile was also assessed by comparing treated and untreated plants within each cultivar. In K326 (PKSe vs PK), 241 differentially accumulated metabolites (DAMs) were identified, with 58 upregulated and 183 downregulated. In Zhongyan 100 (PZSe vs PZ), 266 DAMs were altered, with 25 upregulated and 241 downregulated. Notably, the number of downregulated metabolites vastly outnumbered upregulated ones in both cultivars following selenium application. KEGG enrichment analysis of these DAMs revealed that selenium treatment significantly affected the Phenylpropanoid biosynthesis pathway in both cultivars. Additionally, pathways such as Flavonoid biosynthesis and ABC transporters were enriched in K326, while Purine metabolism was prominently enriched in Zhongyan 100.

### 3.11 Integrated analysis of the transcriptome and metabolome

**Figure 10:**
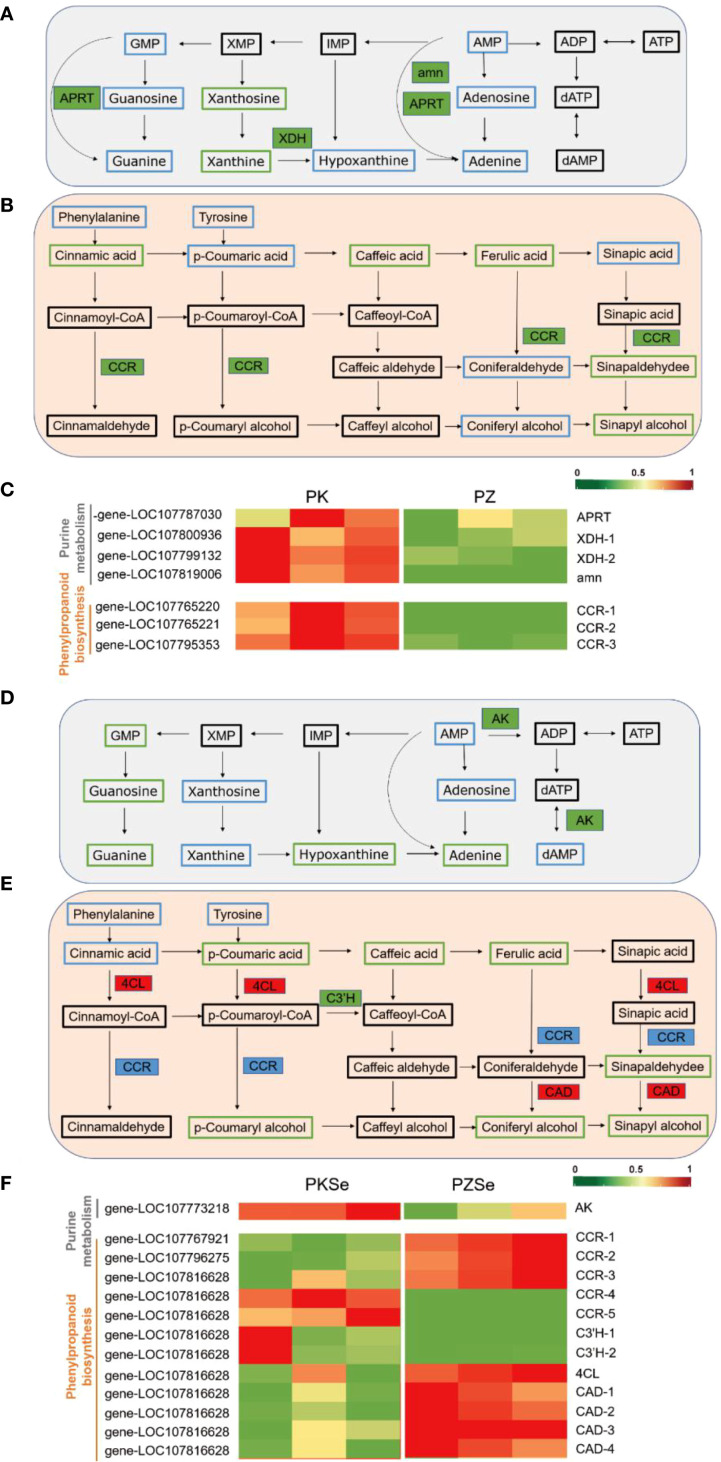
Metabolite pathway map in response to *P. nicotianae* infection in two tobacco cultivars and qPCR validation of DEGs in metabolic pathways. **(A, D)** Purine metabolism pathway. **(B, E)** Phenylpropane biosynthesis pathway. **(C, F)**, Heatmaps of differential gene expression. Green represents downregulation, red represents upregulation, blue indicates that the metabolite was detected but the difference was not significant, and black indicates that the metabolite was not detected. Solid boxes indicate differentially expressed enzymes, with green representing downregulation, and red representing upregulation, and blue representing both up- and downregulation.

Interactions between plants and their pathogens involve complex molecular and metabolic networks. Therefore, integrating metabolomics and transcriptomics is essential for identifying crucial metabolic pathways, key metabolites, and regulatory genes, enabling multiomics cascade analysis at the network level. On the basis of KEGG pathway analysis of both metabolite and gene expression data, coenriched pathway networks were screened, and DEGs and DAMs were mapped to the KEGG database to generate integrated pathway maps (**Figures 10A, B**). Network-level interactions were deciphered by comparing DEGs and DAMs between two tobacco varieties following *P. nicotianae* infection. Notably, both DEGs and DAMs were significantly enriched in purine metabolism and phenylpropanoid biosynthesis pathways across the experimental groups, highlighting their central roles in pathogen response mechanisms.

Integrated transcriptomic and metabolomic analyses revealed that in Zhongyan 100, genes encoding xanthine dehydrogenase (XDH), AMP nucleosidase (AMN), and adenine phosphoribosyltransferase (APRT) within the purine metabolism pathway were significantly downregulated compared with those in K326. Similarly, the levels of the key DMs xanthine nucleoside and xanthine in the PZ group decreased to 33.51% and 36.20% of those in the PK group, respectively. (**Figure 10A**; **Supplementary Table S9**). Additionally, three cinnamoyl-CoA reductase (CCR)-encoding genes in the phenylpropanoid biosynthesis pathway were markedly downregulated, which aligns with the reduced accumulation of phenolic precursors, including cinnamic acid, caffeic acid, ferulic acid, sinapaldehyde, and sinapyl alcohol, in the PZ group (**Figure 10C**). These findings demonstrate that under *P. nicotianae* infection, Zhongyan 100 accumulates substantially lower levels of phenolic acids and flavonoids than K326, suggesting that K326 has higher resistance to tobacco black shank disease because of coordinated regulation of genetic material metabolism, nitrogen homeostasis, ROS scavenging, and lignin synthesis.

Comparative analysis of the PKSe vs. PZSe group revealed that DEGs and metabolites are enriched in the purine metabolism and phenylpropanoid biosynthesis pathways. Distinct from the PK vs PZ group, xanthine and xanthosine in PZSe showed no significant downregulation, whereas guanosine monophosphate (GMP), guanosine, guanine, and hypoxanthine were markedly reduced, accompanied by downregulated expression of adenosine kinase (AK)-encoding genes. In the phenylpropanoid biosynthesis pathway, five genes encoding CCR were differentially expressed (including three genes upregulated and two genes downregulated), with concurrent downregulation of 5-O-(4-coumaroyl)-D-quinate 3’-monooxygenase (C3’H) and upregulation of the 4-coumarate-CoA ligase (4CL) and cinnamyl alcohol dehydrogenase (CAD) genes (**Figures 10D, E**; **Supplementary Table S10**). Metabolite profiling revealed reduced levels of ferulic acid, coumaric acid, coumaroyl-CoA, coniferyl alcohol, sinapaldehyde, and sinapyl alcohol in PZSe. Compared with those in PK vs. PZ, the selective upregulation of phenylpropanoid-related genes contrasted with decreased p-coumaric acid, p-coumaryl alcohol, and coniferyl alcohol levels in PZSe. Notably, caffeic acid, sinapaldehyde, and sinapyl alcohol were consistently downregulated across both groups (**Figure 10F**). These results indicate that selenium induction differentially modulates gene–metabolite networks in tobacco varieties, with Zhongyan 100 exhibiting weaker disease resistance than K326 does. Although selenium upregulated 4CL, CAD, and CCR expression, the ultimate metabolite depletion suggests that selenium preferentially enhances defence mechanisms in the K326 cultivar through pathways extending beyond phenylpropanoid flux.

## 4 Discussion

### 4.1 Selenium exhibits differential regulation in antioxidant systems and membrane stability of resistant and susceptible tobacco varieties to enhance plants resistance against tobacco black shank

Selenium, a critical regulatory factor in plant disease resistance, modulates tobacco resistance to black shank disease through multiple physiological mechanisms, with significant differences observed between cultivars. Comparative analysis of tobacco cultivars with varying resistance levels revealed that the resistant cultivar K326 exhibited substantial physiological advantages over the susceptible cultivar Zhongyan 100 under *Phytophthora parasitica* infection. Specifically, the flavonoid and total phenolic contents of K326 were markedly greater than those of Zhongyan 100. This observation is consistent with the ability of flavonoids to scavenge ROS and thus alleviate oxidative damage (Long et al., 2019). Previous studies have confirmed that flavonoid biosynthesis is directly regulated by *WRKY* transcription factors. For example, the absence of *HvWRKY23* in barley significantly suppresses flavonoid accumulation, leading to reduced resistance to Fusarium head blight (Karre et al., 2019). Furthermore, the high accumulation of phenolic compounds in resistant plants is widely recognised as a key strategy against pathogen invasion, functioning through direct inhibition of pathogen growth or reinforcement of physical barriers in the cell wall (Bakan et al., 2003). Under pathogen stress, K326 not only maintained elevated phenolic levels under pathogen stress but also exhibited higher soluble protein contents and lower malondialdehyde (MDA) levels compared with Zhongyan 100, indicating enhanced membrane system stability specifically in the resistant cultivar. These findings align with observations of rice resistance to *Ustilaginoidea virens* (Kou et al., 2019), suggesting that the synergistic interaction between antioxidant metabolism and membrane protection mechanisms may be a core feature underlying cultivar-specific differences in disease resistance.

Further analysis of the effects of selenium treatment on physiological parameters revealed that exogenous selenium significantly activated the host defence enzyme system, but to differing extents between cultivars. In the resistant cultivar K326, the activities of CAT, POD and SOD peaked at a selenium concentration of 8 mg/L, with values significantly exceeding those of Zhongyan 100, which was accompanied by a more pronounced concomitant reduction in the accumulation of reactive oxygen species (H_2_O_2_ and O_2_^-^). These results collectively demonstrate that the superior resistance of K326 is underpinned by a more efficient ROS scavenging system, which is further potentiated by selenium application. The coordinated enhancement of antioxidant enzymes and the concomitant reduction in ROS levels constitute a classic defence mechanism against biotic stress, effectively minimising oxidative damage to cellular components. These enzymes, as key components of ROS scavenging, mitigate oxidative damage by regulating free radical homeostasis during pathogen invasion (Mittler et al., 2011). Notably, the activation of antioxidant systems was significantly correlated with membrane stability. When relative electrical conductivity was used as an indicator, selenium treatment markedly reduced membrane lipid peroxidation in both cultivars, yet K326 exhibited a more pronounced improvement compared with Zhongyan 100. Concurrently, the accumulation of soluble sugars, which serve as osmoregulatory metabolites, increases with increasing selenium concentration and displays synchronised trends with antioxidant enzyme activities (Elkelish et al., 2019). At 8 mg/L selenium, disease incidence reached its minimum in both cultivars, and relative electrical conductivity was significantly negatively correlated with disease incidence, demonstrating that selenium effectively suppresses pathogen invasion by enhancing membrane integrity and osmoregulatory capacity.

Cell necrosis visualisation via trypan blue staining further validated the superior resistance conferred by selenium in K326 compared with Zhongyan 100 (Strober, 2015). Selenium-treated K326 leaves presented significantly fewer necrotic regions (blue-stained areas) caused by membrane permeability loss than those in Zhongyan 100, indicating that selenium enhanced infection resistance more effectively in the K326 cultivar through the superior maintenance of selective membrane permeability. These results align closely with the electrical conductivity data, highlighting the central role of selenium-mediated membrane protection in the differential disease resistance between cultivars. Intriguingly, although selenium treatment increased antioxidant enzyme activities and soluble sugar levels in Zhongyan 100, its membrane stability remained significantly inferior to that of K326 under equivalent treatment, suggesting that the resistance disparity may originate from holistic molecular regulatory mechanisms intrinsic to each cultivar. The concurrently improved photosynthetic performance (*Pn*, *Gs*, *Tr*) and enhanced CO_2_ utilisation efficiency (lower *Ci*) in K326 further underscore its superior ability to maintain fundamental physiological processes under combined selenium and pathogen treatment. In summary, selenium synergistically enhances tobacco resistance to black shank disease by activating antioxidant systems, optimising membrane stability, and increasing the osmoregulatory capacity, with the resistant cultivar K326 exhibiting significantly greater physiological responses and resultant resistance advantages over the susceptible Zhongyan 100.

### 4.2 Selenium optimises photosynthetic efficiency and sugar metabolism of tobacco resistant variety to improve plants resistance against tobacco black shank

The synergistic regulation of metabolic and transcriptional networks is pivotal for establishing disease resistance during plant-pathogen interactions. Through integrated multiomics analyses, this study systematically revealed the molecular mechanisms by which selenium modulates the distinct defence responses and resultant resistance levels to *Phytophthora parasitica* infection in the tobacco cultivars, K326 and Zhongyan 100. Specifically, selenium differentially regulated phenylpropanoid metabolism, purine metabolism, and photosynthesis-related pathways between cultivars, with K326 exhibiting a more pronounced enhancement in resistance compared with Zhongyan 100.

The phenylpropanoid metabolism pathway is fundamental to plant disease resistance, where its end products, such as lignin, flavonoids, and phenolic acids, fortify cell walls and synthesise antimicrobial compounds to combat pathogens (Dixon and Paiva, 1995; Yoshikawa et al., 2018). The robust upregulation of phenylpropanoid biosynthesis genes and the significant accumulation of lignin precursors (e.g., coniferyl alcohol, sinapyl alcohol) and phenolic acids (e.g., ferulic acid, caffeic acid) in K326 directly translate into the fortification of the cell wall lignin barrier and the production of antifungal metabolites. This dual strategy physically impedes pathogen penetration and chemically inhibits its growth, representing a fundamental layer of plant innate immunity. A critical comparison of the tobacco cultivars K326 and Zhongyan 100 reveals distinct genotypic responses to Se in enhancing resistance to black shank disease, with K326 demonstrating a clear advantage. Untreated Zhongyan 100 exhibited significant downregulation of key phenylpropanoid genes (CCR, CAD, 4CL) compared with the results for K326, leading to substantially lower accumulation of lignin precursors (cinnamyl alcohol, coniferyl alcohol, sinapyl alcohol) and phenolic acids (caffeic acid, ferulic acid). This deficiency compromises cell wall lignification, weakening the physical barrier against pathogen invasion (Rao et al., 2015). Concurrent suppression of flavonoid biosynthesis (e.g., isorhamnetin-7-O-glucoside) further diminishes its ability to scavenge ROS and direct antimicrobial activity (Mierziak et al., 2014). Selenium treatment upregulated 4CL, CCR, and CAD expression levels in both cultivars, yet the resulting metabolic enhancement and consequent resistance boost were markedly superior in K326. Crucially, even with Se induction, Zhongyan 100 maintained significantly lower levels of key lignin monomers (p-coumaryl alcohol, coniferyl alcohol) than did K326, indicating genotype-specific bottlenecks hindering the translation of gene activation into an effective accumulation of defence metabolites. Transcriptomic data suggested potential bottlenecks beyond core biosynthetic genes, including significant downregulation of genes encoding ABC transporters (e.g., ABCG15, ABCG25) involved in phenolic compound translocation and key transcriptional regulators (e.g., MYB4, MYB7) known to suppress phenylpropanoid flux in Zhongyan 100 compared to K326 under Se treatment (PKSe vs PZSe). Conversely, K326 leveraged the Se treatment more effectively, maintaining a higher phenylpropanoid flux through robust upregulation of 4CL and CAD, accompanied by specific accumulation of antimicrobial phenolic acids (ferulic acid, caffeic acid) that increase both cell wall lignification and antimicrobial compound synthesis. A key advantage for K326 was the Se-induced elevation of sinapyl alcohol, a metabolite documented to directly disrupt pathogen membrane integrity (Ortiz and Sansinenea, 2023), demonstrating effective transcriptional control over critical defence metabolites in this cultivar. Therefore, the differential response underscores the genotype-dependent nature of selenium’s regulation of phenylpropanoid metabolism, with K326 achieving superior pathogen resistance through its enhanced capacity for coordinated gene expression and the consequential synthesis of protective metabolites.

Purinergic metabolism not only supplies energy carriers for plant defence responses but also modulates disease resistance through nitrogen recycling and the regulation of ROS homeostasis (Irani and Todd, 2016). Our findings directly support this mechanism. The sustained purine metabolism in K326, characterised by the maintained expression of genes like XDH and APRT, ensures a steady supply of ATP and metabolic precursors. This reliable energy provision is crucial for fuelling energy-demanding defence responses, such as the massive biosynthesis of defensive compounds and the maintenance of ion gradients across membranes during pathogen attack. When comparing varietal responses to selenium under pathogen stress, Zhongyan 100 displayed downregulated expression of key purine metabolism genes, including XDH and APRT, leading to significantly reduced levels of metabolites such as xanthosine and xanthine. XDH, a critical enzyme in purine catabolism, facilitates the conversion of xanthine to allantoin; its diminished activity in Zhongyan 100 not only impaired nitrogen salvage but also reduced the ROS-scavenging capacity (Urarte et al., 2015). Concurrently, suppressed APRT expression restricted AMP synthesis, disrupting cellular energy dynamics (ATP/ADP cycling) and cytokinin signalling. These metabolic perturbations compromised nitrogen homeostasis and energy supply in Zhongyan 100 under pathogen stress, exacerbating metabolic dysregulation (Baena-Gonzalez and Sheen, 2008; Liu et al., 2023). Selenium treatment dynamically reprogrammed purine metabolism, revealing a distinct advantage in K326 over Zhongyan 100 for disease resistance. In K326, despite decreased GMP and guanine levels, selenium upregulated AK expression to enhance adenosine-to-AMP conversion, thereby promoting energy production crucial for pathogen defence (Regierer et al., 2002). Furthermore, selenium induced the upregulation of glutathione peroxidase (GPX) in K326, promoting reduced glutathione (GSH) accumulation and establishing an efficient ROS-scavenging system (Herbette et al., 2006). In contrast, Zhongyan 100 failed to restore purine metabolic flux under selenium supplementation, as evidenced by sustained decreases in GMP and guanine, which impeded nucleic acid synthesis and reduced metabolic efficiency in this variety. This discrepancy highlights the genotype-specific capacity of K326 to utilise selenium to optimise the balance between purine catabolism and anabolism. Crucially, selenium established a more efficient energy allocation system in K326 through coordinated metabolic adjustments, providing the metabolic foundation for its enhanced black shank disease resistance compared with Zhongyan 100.

Photosynthesis and carbohydrate metabolism constitute the core of plant energy provision, and their efficiency directly influences disease resistance (Rojas et al., 2014). When comparing varietal responses to selenium under pathogen stress, K326 demonstrated significant enrichment of genes associated with carbon fixation (e.g., ribulose-1,5-bisphosphate carboxylase/oxygenase) and photosynthetic pigment biosynthesis, alongside the activation of vitamin B6 metabolism. As vitamin B6 is a coenzyme involved in gluconeogenesis and antioxidative reactions, enhanced vitamin B6 metabolism likely sustains Calvin cycle efficiency to ensure the supply of photosynthetic products and provide carbon skeletons for defence compounds (Tambasco-Studart et al., 2005). Metabolomic profiling of K326 further revealed the selenium-induced accumulation of osmoregulatory substances (D-fructose, D-mannitol), which stabilise membrane integrity and mitigate osmotic stress (Nawaz et al., 2016). The enrichment of carotenoid biosynthesis pathways suggests that selenium may reduce photoinhibition by reinforcing photoprotective mechanisms in this variety. Carotenoids act as chloroplast antioxidants, quenching pathogen-induced ROS overaccumulation and preventing oxidative damage to photosystem II (PSII) (Müller-Moulé et al., 2002). Notably, elevated fructose and glucose derivatives (e.g., 5-O-ferulic acid) in K326 potentially enhance defence responses via jasmonic acid (JA) signalling activation (Tauzin and Giardina, 2014). In contrast, Zhongyan 100 showed limited accumulation of photosynthesis-related metabolites (e.g., chlorophyll precursors) under selenium treatment despite the differential expression of carbohydrate metabolism genes, resulting in inadequate photoprotection and exacerbated oxidative damage (Tanaka and Tanaka, 2007). Furthermore, selenium-induced metabolic reprogramming in K326 likely establishes a positive feedback loop by activating hexokinase-mediated signalling, which coordinates ROS signalling with defence gene expression (Bolouri et al., 2012). However, Zhongyan 100 failed to integrate its metabolic and transcriptional responses effectively because of inefficient sugar signal transduction, underscoring the superior capacity of K326 to leverage selenium for enhanced black shank disease resistance compared with Zhongyan 100.

Integrated transcriptomic and metabolomic analyses reveal that selenium differentially enhances disease resistance across tobacco varieties through a distinct coordination between purine metabolism and the phenylpropanoid pathway. In cultivar K326, selenium-stabilised purine metabolism (e.g., GMP retention) provides sustained energy and reducing power (NADPH) for phenylpropanoid biosynthesis. Concurrently, activated phenylpropanoid metabolism synthesises phenolic acids that inhibit pathogen-derived enzymes, establishing a “metabolism–defence” synergistic loop. Enhanced carbohydrate metabolism not only supplies carbon skeletons but also generates signalling molecules (e.g., sucrose derivatives) to regulate defence-related transcription factors (MYB, WRKY), amplifying selenium-mediated effects. In contrast, the cultivar Zhongyan 100 exhibits compromised purine metabolism that limits ATP/NADPH availability, constraining phenylpropanoid flux. Its inefficient carbohydrate metabolism further limits carbon allocation to defence compounds, restricting selenium responses to transcriptional regulation without phenotypic resistance manifestation. This metabolic disparity underscores the superior capacity of K326 to utilise selenium for constructing a multilayered defence network against black shank disease compared with Zhongyan 100.

Overall, exogenous selenium confers superior black shank disease resistance in the resistant cultivar K326 compared with the susceptible Zhongyan 100 by specifically enhancing key metabolic pathways. Primarily within phenylpropanoid biosynthesis, selenium significantly upregulates genes such as 4CL, CCR, and CAD in K326, boosting lignin precursor synthesis and phenolic acid production to establish a dual physical and chemical defence system. Concurrently in purine metabolism, selenium sustains high expression levels of genes such as XDH and APRT in K326, maintaining purine homeostasis to ensure robust energy provision and support defence responses. Conversely, inherent metabolic deficiencies in Zhongyan 100 limit its ability to effectively integrate selenium regulation, resulting in comparatively weaker disease resistance improvement despite identical treatment. These findings elucidate the metabolic basis for genotype-specific selenium-induced resistance and provide critical insights for precision selenium application in protecting resistant cultivars against tobacco black shank disease.

## 5 Conclusions

Foliar selenium application significantly reduced disease incidence in both cultivars but conferred superior resistance in K326 compared with Zhongyan 100. The K326 cultivar exhibited greater induction of antioxidant enzyme activities, higher plant membrane stability, more pronounced differential expression of genes linked to phenylpropanoid biosynthesis, purine metabolism and photosynthesis, as well as higher accumulation of defence metabolites, including phenolic acids, flavonoids and osmolytes. In addition, selenium synergistically bolstered resistance in K326 by effectively coordinating phenylpropanoid flux with purine homeostasis, optimising photosynthetic efficiency and sugar metabolism, whereas Zhongyan 100 exhibited metabolic bottlenecks that limited the translation of transcriptional activation into functional metabolite accumulation.

## Data Availability

The datasets presented in this study are publicly available. This data can be found here: https://www.ncbi.nlm.nih.gov/sra, accession number PRJNA913648.
