# Supplementary material for: Integrated transcriptomic and metabolomic analysis of resistant and susceptible Nicotiana tabacum L. reveals the mechanisms of selenium-induced disease resistance to Phytophthora nicotianae
Source: Front Plant Sci. 2025 Oct 8;16:1663346. doi: 10.3389/fpls.2025.1663346 (PMC12542911; doi:10.3389/fpls.2025.1663346)
Supplement: Supplementary file 1 [file Supplementaryfile1.docx]

Supplementary Material

**Table S1 Specific primer sequences for qRT-PCR**

| Gene ID | Forward | Reverse |
| --- | --- | --- |
| AM588_10003354 | GAACTTCGTATCGCAACC | GCTTCAATCAGCTCCTGTAA |
| AM588_10000227 | ACTGGATACGGTAGACAGACAC | CAATGCTGCTGACCTTGC |
| AM588_10009779 | GCGTAGTAGTCCTCCCTCA | TCGCTGGATGAAATGGTAT |
| AM588_10009846 | TTTAGCGAAGCGAGGGAC | CGAACACCCGCCTACTTT |
| AM588_10004717 | GGCACCGCAGATAACAAA | GCCTCGTGATTCCCTACA |
| AM588_10009072 | CACGCTCGCCAATCTCAG | CAAGGTTGTGGTGCTATGC |
| AM588_10009073 | TCATGGAACATCAGCCGTAC | TCGCCAATCTCAGCCTCT |
| AM588_10006215 | AATGCCCAGCGACGAAGA | GTGCTCAGATTGCCCAGA |
| AM588_10002231 | GAAGCGTGTTCAGGTTATT | GCTCCATTCTGCTCGTCT |
| AM588_10009761 | CTGCCACTTCTGGTTCCT | GACCTTGTTAAGCGTTTCC |
| AM588_10004044 | AGTGCTTGGCGAGGATAA | GCCAGTGCCTACTATGACG |
| *PpUBC* | GAGCCCTGCGTTGACTATCT | TACTTGGCGGTCCATTCG |

**Table S2 Incidence analysis of four varieties of *P. nicotianae* infected**

| Species | Incidence number | Total | Incidence rate |
| --- | --- | --- | --- |
| K326 | 37 | 50 | 74.00% |
| Hongda | 44 | 50 | 88.00% |
| Zhongyan 100 | 49 | 50 | 98.00% |
| Changbohuang | 47 | 50 | 94.00% |

**Table S3 Statistics on the quality of sequencing data for each treatment sample**

| Sample | Raw Reads | Clean Reads | Clean Base(G) | Error Rate(%) | Q20(%) | Q30(%) | GC Content(%) |
| --- | --- | --- | --- | --- | --- | --- | --- |
| PK-1 | 46518800 | 42477954 | 6.37 | 0.02 | 98.38 | 94.94 | 41.95 |
| PK-2 | 44727416 | 41510850 | 6.23 | 0.03 | 98.01 | 94.03 | 42.11 |
| PK-3 | 43966966 | 40747624 | 6.11 | 0.03 | 98.14 | 94.3 | 42.07 |
| PKSe-1 | 43577094 | 41377848 | 6.21 | 0.03 | 97.98 | 93.94 | 42.45 |
| PKSe-2 | 45868240 | 43616940 | 6.54 | 0.03 | 97.85 | 93.68 | 42.85 |
| PKSe-3 | 45792470 | 43529474 | 6.53 | 0.03 | 97.87 | 93.70 | 42.76 |
| PZ-1 | 46866952 | 43225414 | 6.48 | 0.03 | 97.96 | 93.91 | 41.91 |
| PZ-2 | 43310424 | 39969088 | 6.00 | 0.03 | 98.08 | 94.16 | 41.8 |
| PZ-3 | 49738528 | 48818480 | 7.32 | 0.03 | 97.79 | 93.61 | 42.34 |
| PZSe-1 | 45540216 | 42433966 | 6.37 | 0.03 | 97.80 | 93.58 | 42.29 |
| PZSe-2 | 46725274 | 43745418 | 6.56 | 0.03 | 97.97 | 93.95 | 42.08 |
| PZSe-3 | 47630882 | 44027320 | 6.6 | 0.03 | 97.87 | 93.76 | 42.05 |

**Table S4 Statistics on the quality of sequencing data for each treatment sample**

| Sample | Total Reads | Reads mapped | Unique mapped | Multi mapped |
| --- | --- | --- | --- | --- |
| PK-1 | 42537588 | 40813584(95.95%) | 39595996(93.08%) | 1677074(2.86%) |
| PK-2 | 41580966 | 39817681(95.76%) | 38675430(93.01%) | 1503312(2.75%) |
| PK-3 | 40804778 | 39395447(96.55%) | 38293599(93.85%) | 1366778(2.70%) |
| PKSe-1 | 41436780 | 39444072(95.19%) | 38376062(92.61%) | 1350549(2.58%) |
| PKSe-2 | 43734678 | 41310280(94.46%) | 39835101(91.08%) | 2261491(3.37%) |
| PKSe-3 | 43602146 | 41872386(96.03%) | 40627502(93.18%) | 1696731(2.86%) |
| PZ-1 | 43225414 | 41126064(95.14%) | 39921009(92.36%) | 1205055(2.79%) |
| PZ-2 | 39969088 | 38282009(95.78%) | 37229628(93.15%) | 1052381(2.63%) |
| PZ-3 | 48818480 | 45976140(94.18%) | 44686196(91.54%) | 1289944(2.64%) |
| PZSe-1 | 42433966 | 40170523(94.67%) | 38885907(91.64%) | 1284616(3.03%) |
| PZSe-2 | 43745418 | 41893044(95.77%) | 40700179(93.04%) | 1192865(2.73%) |
| PZSe-3 | 44027320 | 41810466(94.96%) | 40578898(92.17%) | 1231568(2.80%) |

**Table S5 Differential metabolites downregulated in the PK vs PZ group**

| Compounds | Primary classification | PK | PZ | VIP | log2FC |
| --- | --- | --- | --- | --- | --- |
| Caffeoylcholine | Alkaloids | 20271 | 9 | 1.94 | -11.14 |
| 6,7-Dihydroxy-4-methylcoumarin | Lignans and Coumarins | 19000 | 9 | 1.96 | -11.04 |
| Sinapyl alcohol | Phenolic acids | 10588 | 9 | 1.92 | -10.20 |
| Eriodictyol-7-O-glucoside | Flavonoids | 5044 | 9 | 1.94 | -9.13 |
| 4-Hydroxyacetophenone | Phenolic acids | 463706 | 40025 | 1.46 | -3.53 |
| 5'-Glucosyloxyjasmanic acid | Phenolic acids | 942317 | 115302 | 1.30 | -3.03 |
| Cinnamic acid | Phenolic acids | 61692 | 10358 | 1.41 | -2.57 |
| β-Nicotyrine | Alkaloids | 656213 | 117377 | 1.81 | -2.48 |
| 2-Hydroxycinnamic acid | Organic acids | 1321783 | 270697 | 1.04 | -2.29 |
| Kaempferol-3-O-sophoroside-7-O-rhamnoside | Flavonoids | 7503 | 1540 | 1.27 | -2.28 |
| Sinapinaldehyde | Phenolic acids | 410003 | 92562 | 1.42 | -2.15 |
| L-Ascorbic acid (Vitamin C) | Others | 15942 | 3864 | 1.10 | -2.04 |
| 3,4-Dihydroxyacetophenone | Phenolic acids | 125311 | 31116 | 1.67 | -2.01 |
| Kaempferol-3-O-neohesperidoside-7-O-glucoside | Flavonoids | 5036 | 1263 | 1.46 | -2.00 |
| 10-Heptadecenoic Acid | Lipids | 175879 | 44993 | 1.55 | -1.97 |
| Jasmonic acid | Organic acids | 74346 | 19763 | 1.80 | -1.91 |
| Acetovanillone | Phenolic acids | 23382 | 6220 | 1.36 | -1.91 |
| Cotinine-glucoside | Alkaloids | 94730 | 25720 | 1.88 | -1.88 |
| Nicotine-N-glucuronide | Alkaloids | 85848 | 23721 | 1.75 | -1.86 |
| Androsin | Others | 73355 | 21290 | 1.41 | -1.78 |
| 2-(Formylamino)benzoic acid | Phenolic acids | 203170 | 66390 | 1.01 | -1.61 |
| Cotinine | Alkaloids | 86414 | 28629 | 1.39 | -1.59 |
| Ferulic acid | Phenolic acids | 197583 | 66099 | 1.09 | -1.58 |
| Xanthosine | Nucleotides and Derivatives | 1334263 | 447073 | 1.09 | -1.58 |
| Eleutheroside B1 | Lignans and Coumarins | 159150 | 53656 | 1.70 | -1.57 |
| Muconic acid | Organic acids | 7071333 | 2435067 | 1.68 | -1.54 |
| 6-Hydroxy-N-methylmyosmine | Alkaloids | 2602467 | 923577 | 1.35 | -1.49 |
| Pentadecanoic Acid | Lipids | 373463 | 132791 | 1.34 | -1.49 |
| Caffeic acid | Phenolic acids | 6460367 | 2299100 | 1.78 | -1.49 |
| 4-O-Sinapoylquinic acid | Phenolic acids | 4580 | 1640 | 1.15 | -1.48 |
| Xanthine | Nucleotides and Derivatives | 768213 | 278112 | 1.05 | -1.47 |
| Benzoic acid | Phenolic acids | 18740 | 6899 | 1.36 | -1.44 |
| 7S,8S-DiHODE | Lipids | 6279 | 2322 | 1.25 | -1.44 |
| Acetryptine | Alkaloids | 11130 | 4264 | 1.25 | -1.38 |
| Pomolic acid | Terpenoids | 10434333 | 4063833 | 1.60 | -1.36 |
| 3'-Hydroxycotinine | Alkaloids | 40346 | 15756 | 1.36 | -1.36 |
| 3-Aminosalicylic acid | Phenolic acids | 446393 | 176305 | 1.38 | -1.34 |
| 1-Caffeoylquinic acid | Phenolic acids | 2255733 | 895750 | 1.82 | -1.33 |
| Esculetin | Lignans and Coumarins | 551940 | 222987 | 1.73 | -1.31 |
| Geniposide | Terpenoids | 136250 | 56489 | 1.64 | -1.27 |
| Ganoderic Acid DM | Terpenoids | 55169 | 22895 | 1.58 | -1.27 |
| 1-Methyladenosine | Nucleotides and Derivatives | 8827 | 3669 | 1.54 | -1.27 |
| Nepetin | Flavonoids | 13383 | 5563 | 1.54 | -1.27 |
| Hederagenin | Terpenoids | 10941700 | 4556067 | 1.59 | -1.26 |
| Nicotiflorin | Flavonoids | 38215 | 16089 | 1.47 | -1.25 |
| Calycosin-7-O-glucoside | Flavonoids | 963983 | 406507 | 1.46 | -1.25 |
| Caffeoylnicotinoyltartaric acid | Phenolic acids | 85654 | 36970 | 1.83 | -1.21 |
| L-Cysteine | Amino acids and Derivatives | 345337 | 151697 | 1.13 | -1.19 |
| Isofraxidin | Lignans and Coumarins | 122955 | 54113 | 1.37 | -1.18 |
| 2'-Deoxyinosine-5'-monophosphate | Nucleotides and Derivatives | 890570 | 394741 | 1.29 | -1.17 |
| Oxyphyllol A | Terpenoids | 9740 | 4339 | 1.28 | -1.17 |
| Luteolin-7-O-rutinoside | Flavonoids | 40473 | 18114 | 1.31 | -1.16 |
| Syringaldehyde | Phenolic acids | 55128 | 24673 | 1.24 | -1.16 |
| Methylenesuccinic acid | Organic acids | 21345 | 9589 | 1.35 | -1.15 |
| 2-Hydroxyisocaproic acid | Organic acids | 120823 | 54776 | 1.18 | -1.14 |
| Penstemonoside | Terpenoids | 48569 | 22360 | 1.11 | -1.12 |
| N-Oleoylethanolamine | Alkaloids | 36079 | 16853 | 1.79 | -1.10 |
| 2-Acetyl-2-Hydroxybutanoic Acid | Organic acids | 45358 | 21387 | 1.21 | -1.08 |
| N-Acetyl-D-mannosamine | Others | 30621 | 14464 | 1.62 | -1.08 |
| p-Coumaroyltyramine | Alkaloids | 2087833 | 1025260 | 1.71 | -1.03 |
| 3-(4-Hydroxyphenyl)-propionic acid | Phenolic acids | 29984 | 14755 | 1.51 | -1.02 |
| β-Hydroxyisovaleric acid | Organic acids | 419663 | 207390 | 1.26 | -1.02 |
| Biorobin | Flavonoids | 157117 | 77767 | 1.31 | -1.01 |

**Table S6 Differential metabolites upregulated in the PK vs PZ group**

| Compounds | Primary Classification | PK | PZ | VIP | log2FC |
| --- | --- | --- | --- | --- | --- |
| L-Aspartic acid-O-diglucoside | Amino acids and derivatives | 1403593 | 10734033 | 1.90 | 2.93 |
| L-Proline | Amino acids and derivatives | 982903 | 5889900 | 1.93 | 2.58 |
| D-Arabinose | Others | 12814 | 44361 | 1.12 | 1.79 |
| D-Sorbitol | Others | 6143 | 21218 | 1.29 | 1.79 |
| D-Fructose | Others | 1288713 | 4439733 | 1.21 | 1.78 |
| Kaempferol-4'-O-glucoside | Flavonoids | 22700 | 77736 | 1.57 | 1.78 |
| D-Glucose | Others | 1283527 | 3999933 | 1.20 | 1.64 |
| Luteolin-7-O-rutinoside-5-O-rhamnoside | Flavonoids | 2098 | 6002 | 1.38 | 1.52 |
| Solatriose | Others | 74402 | 209213 | 1.35 | 1.49 |
| Anthranilate-1-O-Sophoroside | Phenolic acids | 19421 | 53639 | 1.06 | 1.47 |
| Cynaroside | Flavonoids | 28613 | 74337 | 1.60 | 1.38 |
| 3-Hydroxy-3-methylpentane-1,5-dioic acid | Amino acids and derivatives | 2829033 | 7340333 | 1.61 | 1.38 |
| 5-O-Feruloylquinic acid | Phenolic acids | 3856 | 9846 | 1.18 | 1.35 |
| Furanofructosyl-α-D-(3-mustard acyl)glucoside | Phenolic acids | 8277 | 21083 | 1.80 | 1.35 |
| D-Glucoronic acid | Others | 66194 | 156387 | 1.81 | 1.24 |
| 2-Aminopurine | Nucleotides and derivatives | 58824 | 136267 | 1.89 | 1.21 |
| Astragalin | Flavonoids | 27383 | 63023 | 1.31 | 1.20 |
| Luteolin-4'-O-glucoside | Flavonoids | 32653 | 74909 | 1.25 | 1.20 |
| Hinokitiol | Terpenoids | 6357 | 13515 | 1.06 | 1.09 |
| Cyclic 3',5'-Adenylic acid | Nucleotides and derivatives | 51615 | 108706 | 1.79 | 1.07 |
| Trigonelline | Alkaloids | 633170 | 1323567 | 1.47 | 1.06 |
| Isomaltulose | Others | 106117 | 221295 | 1.01 | 1.06 |
| 2-Methylsuccinic acid | Organic acids | 436957 | 887973 | 1.60 | 1.02 |
| Epipinoresinol | Lignans and coumarins | 3305 | 6636 | 1.72 | 1.01 |

**Table S7 Differential metabolites downregulated in the PKSe vs PZSe group**

| Compounds | Primary Classification | PKSe | PZSe | VIP | log2FC |
| --- | --- | --- | --- | --- | --- |
| Caffeoylcholine | Alkaloids | 508604 | 9 | 1.68 | -15.79 |
| Sinapyl alcohol | Phenolic Acids | 15610 | 9 | 1.74 | -10.76 |
| 6,7-Dihydroxy-4-methylcoumarin | Lignans and Coumarins | 12110 | 9 | 1.74 | -10.39 |
| Kaempferol-3-O-neohesperidoside-7-O-glucoside | Flavonoids | 12049 | 9 | 1.74 | -10.39 |
| p-Coumaric acid | Phenolic Acids | 11923 | 9 | 1.74 | -10.37 |
| p-Coumaryl alcohol | Phenolic Acids | 9756 | 9 | 1.74 | -10.08 |
| Daphnetin | Lignans and Coumarins | 5105 | 9 | 1.24 | -9.15 |
| LysoPE 15:1(2n isomer) | Lipids | 5005 | 9 | 1.73 | -9.12 |
| Tiliroside | Flavonoids | 1826 | 9 | 1.73 | -7.66 |
| LysoPC 20:4 | Lipids | 760 | 9 | 1.71 | -6.40 |
| 3-O-(2-O-Acetyl-glucosyl)oleanolic acid | Terpenoids | 363155 | 16354 | 1.47 | -4.47 |
| 9,12,13-Trihydroxy-10,15-octadecadienoic acid | Lipids | 275269 | 12618 | 1.37 | -4.45 |
| 9-Hydroxy-13-oxo-10-octadecenoic Acid | Lipids | 450719 | 21136 | 1.40 | -4.41 |
| 1-α-Linolenoyl-glycerol-3-O-glucoside | Lipids | 27202 | 1280 | 1.40 | -4.41 |
| 2-α-Linolenoyl-glycerol-1,3-di-O-glucoside | Lipids | 61915 | 3270 | 1.48 | -4.24 |
| 2-α-Linolenoyl-glycerol-1-O-glucoside | Lipids | 71265 | 4520 | 1.54 | -3.98 |
| 9,10,13-Trihydroxy-11-Octadecenoic Acid | Lipids | 1173583 | 81816 | 1.30 | -3.84 |
| 1-α-Linolenoyl-glycerol-2,3-di-O-glucoside | Lipids | 52927 | 3857 | 1.47 | -3.78 |
| Kaempferol-3-O-sophoroside-7-O-rhamnoside | Flavonoids | 12503 | 948 | 1.55 | -3.72 |
| 9,10,11-Trihydroxy-12-octadecenoic acid | Lipids | 88976 | 8010 | 1.33 | -3.47 |
| 1-Linoleoylglycerol-2,3-di-O-glucoside | Lipids | 25363 | 2438 | 1.55 | -3.38 |
| 13S-Hydroperoxy-9Z,11E-octadecadienoic acid | Lipids | 115877 | 12738 | 1.29 | -3.19 |
| LysoPE 15:1 | Lipids | 30254 | 3771 | 1.62 | -3.00 |
| 2-Linoleoylglycerol-1,3-di-O-glucoside | Lipids | 21178 | 2691 | 1.37 | -2.98 |
| 1-Linoleoyl-sn-glycerol-diglucoside | Lipids | 19168 | 2465 | 1.48 | -2.96 |
| 1-Oleoyl-Sn-Glycerol | Lipids | 20812 | 2775 | 1.43 | -2.91 |
| Luteolin-7-O-rutinoside | Flavonoids | 67633 | 9126 | 1.59 | -2.89 |
| LysoPE 17:1 | Lipids | 53733 | 7597 | 1.64 | -2.82 |
| LysoPC 16:0(2n isomer) | Lipids | 743830 | 105252 | 1.49 | -2.82 |
| Dopamine | Alkaloids | 24792 | 3557 | 1.50 | -2.80 |
| Lauric acid | Lipids | 248445 | 36878 | 1.29 | -2.75 |
| Biorobin | Flavonoids | 222367 | 33946 | 1.58 | -2.71 |
| LysoPC 19:0 | Lipids | 2783 | 426 | 1.28 | -2.71 |
| LysoPC 16:1(2n isomer) | Lipids | 199635 | 31056 | 1.60 | -2.68 |
| LysoPC 15:0(2n isomer) | Lipids | 31622 | 4963 | 1.46 | -2.67 |
| LysoPC 16:1 | Lipids | 196758 | 32121 | 1.57 | -2.61 |
| LysoPE 17:0 | Lipids | 15040 | 2459 | 1.53 | -2.61 |
| LysoPE 18:3 | Lipids | 1103703 | 183337 | 1.59 | -2.59 |
| LysoPE 18:1 | Lipids | 248260 | 41273 | 1.69 | -2.59 |
| Kaempferol-3-O-glucoside-7-O-rhamnoside | Flavonoids | 193847 | 32870 | 1.51 | -2.56 |
| Nicotiflorin | Flavonoids | 54078 | 9248 | 1.59 | -2.55 |
| LysoPE 18:1(2n isomer) | Lipids | 259513 | 44648 | 1.57 | -2.54 |
| 2-Linoleoylglycerol-1-O-glucoside | Lipids | 6699 | 1154 | 1.54 | -2.54 |
| Guaijaverin | Flavonoids | 6371 | 1107 | 1.59 | -2.53 |
| LysoPE 15:0 | Lipids | 48829 | 8546 | 1.60 | -2.51 |
| LysoPC 15:1 | Lipids | 30431 | 5383 | 1.52 | -2.50 |
| Guanosine 3',5'-cyclic monophosphate | Nucleotides and Derivatives | 101177 | 18299 | 1.43 | -2.47 |
| LysoPC 18:1 | Lipids | 2789567 | 509510 | 1.56 | -2.45 |
| LysoPE 20:2(2n isomer) | Lipids | 14731 | 2692 | 1.56 | -2.45 |
| LysoPE 18:2 | Lipids | 36919 | 6782 | 1.58 | -2.44 |
| Isofraxidin | Lignans and Coumarins | 213823 | 40360 | 1.64 | -2.41 |
| LysoPE 16:1(2n isomer) | Lipids | 33042 | 6259 | 1.45 | -2.40 |
| Kaempferitrin | Flavonoids | 8415 | 1597 | 1.40 | -2.40 |
| LysoPC 19:2(2n isomer) | Lipids | 25400 | 4855 | 1.30 | -2.39 |
| Nicotine-N-glucuronide | Alkaloids | 69984 | 13381 | 1.50 | -2.39 |
| LysoPC 18:1(2n isomer) | Lipids | 3011067 | 587283 | 1.59 | -2.36 |
| Kaempferol-3-O-neohesperidoside | Flavonoids | 186680 | 37411 | 1.51 | -2.32 |
| 1-Linoleoylglycerol-3-O-glucoside | Lipids | 6798 | 1383 | 1.38 | -2.30 |
| Fraxidin (8-Hydroxy-6,7-dimethoxycoumarin) | Lignans and Coumarins | 85032 | 17408 | 1.66 | -2.29 |
| LysoPE 16:0(2n isomer) | Lipids | 4561667 | 941430 | 1.57 | -2.28 |
| LysoPE 16:0 | Lipids | 4583033 | 963267 | 1.54 | -2.25 |
| L-Glutamic acid-O-glycoside | Amino Acids and Derivatives | 59482 | 12536 | 1.49 | -2.25 |
| LysoPE 15:0(2n isomer) | Lipids | 43694 | 9230 | 1.60 | -2.24 |
| LysoPE 18:2(2n isomer) | Lipids | 316173 | 68087 | 1.43 | -2.22 |
| LysoPE 18:3(2n isomer) | Lipids | 7131400 | 1539727 | 1.53 | -2.21 |
| LysoPC 18:0(2n isomer) | Lipids | 72433 | 16026 | 1.45 | -2.18 |
| LysoPE 20:2 | Lipids | 23806 | 5387 | 1.62 | -2.14 |
| LysoPC 18:3 | Lipids | 9630 | 2229 | 1.49 | -2.11 |
| 6-Hydroxy-N-methylmyosmine | Alkaloids | 759847 | 179840 | 1.33 | -2.08 |
| LysoPE 18:0 | Lipids | 256807 | 62029 | 1.53 | -2.05 |
| LysoPE 14:0(2n isomer) | Lipids | 8117 | 1964 | 1.56 | -2.05 |
| Nicotinate D-ribonucleoside | Others | 2689383 | 651920 | 1.25 | -2.04 |
| 3'-Hydroxycotinine | Alkaloids | 18062 | 4408 | 1.54 | -2.03 |
| LysoPE 14:0 | Lipids | 8115 | 1994 | 1.53 | -2.03 |
| LysoPC 15:0 | Lipids | 131184 | 32229 | 1.55 | -2.03 |
| LysoPG 16:0 | Lipids | 910230 | 224493 | 1.38 | -2.02 |
| Punicic acid | Lipids | 349307 | 86625 | 1.32 | -2.01 |
| LysoPC 20:2 | Lipids | 29700 | 7457 | 1.60 | -1.99 |
| Uridine | Nucleotides and Derivatives | 2335940 | 592073 | 1.15 | -1.98 |
| γ-Aminobutyric acid | Organic Acids | 1089133 | 279077 | 1.43 | -1.96 |
| LysoPE 17:1(2n isomer) | Lipids | 11794 | 3027 | 1.54 | -1.96 |
| Cytidine | Nucleotides and Derivatives | 536880 | 137850 | 1.37 | -1.96 |
| Cytarabine | Nucleotides and Derivatives | 339573 | 87615 | 1.27 | -1.95 |
| Cotinine | Alkaloids | 21835 | 5674 | 1.00 | -1.94 |
| LysoPC 16:0 | Lipids | 12143867 | 3174300 | 1.52 | -1.94 |
| LysoPC 17:0(2n isomer) | Lipids | 311617 | 84623 | 1.52 | -1.88 |
| LysoPC 17:0 | Lipids | 206797 | 56873 | 1.65 | -1.86 |
| LysoPE 20:3(2n isomer) | Lipids | 9123 | 2607 | 1.60 | -1.81 |
| LysoPC 18:0 | Lipids | 1455940 | 418930 | 1.53 | -1.80 |
| Adenine | Nucleotides and Derivatives | 11861267 | 3444300 | 1.26 | -1.78 |
| 9,12-Octadecadien-6-Ynoic Acid | Lipids | 8407 | 2442 | 1.40 | -1.78 |
| LysoPC 18:2 | Lipids | 20262 | 5909 | 1.42 | -1.78 |
| LysoPC 19:2 | Lipids | 8513 | 2514 | 1.19 | -1.76 |
| N',N'',N'''-p-Coumaroyl-cinnamoyl-caffeoyl spermidine | Alkaloids | 12852 | 3871 | 1.48 | -1.73 |
| Guanine | Nucleotides and Derivatives | 313920 | 94862 | 1.59 | -1.73 |
| 1-Methyladenosine | Nucleotides and Derivatives | 12323 | 3784 | 1.43 | -1.70 |
| LysoPC 18:2(2n isomer) | Lipids | 236530 | 73001 | 1.32 | -1.70 |
| Guanosine | Nucleotides and Derivatives | 3413267 | 1061307 | 1.26 | -1.69 |
| Eleutheroside B1 | Lignans and Coumarins | 212513 | 66280 | 1.42 | -1.68 |
| LysoPG 16:1 | Lipids | 6578 | 2060 | 1.13 | -1.68 |
| LysoPE 20:3 | Lipids | 9836 | 3081 | 1.65 | -1.67 |
| Aucubin | Terpenoids | 6930 | 2186 | 1.20 | -1.66 |
| LysoPC 17:1 | Lipids | 79742 | 25474 | 1.40 | -1.65 |
| Cotinine-glucoside | Alkaloids | 71777 | 23011 | 1.50 | -1.64 |
| Geniposide | Terpenoids | 152723 | 49009 | 1.51 | -1.64 |
| 13S-Hydroxy-9Z,11E,15Z-octadecatrienoic acid | Lipids | 53937 | 17663 | 1.30 | -1.61 |
| Coniferyl alcohol | Phenolic Acids | 37521 | 12293 | 1.26 | -1.61 |
| N,N-Dimethylglycine | Amino Acids and Derivatives | 702790 | 231053 | 1.64 | -1.60 |
| Indole-5-carboxylic acid | Alkaloids | 62021 | 20484 | 1.07 | -1.60 |
| LysoPC 14:0 | Lipids | 24673 | 8217 | 1.47 | -1.59 |
| Esculetin | Lignans and Coumarins | 331390 | 110836 | 1.56 | -1.58 |
| Ayapin | Lignans and Coumarins | 108449 | 37544 | 1.51 | -1.53 |
| Oleamide | Lipids | 24652 | 8556 | 1.24 | -1.53 |
| O-rhamnoside | Others | 13595 | 4724 | 1.47 | -1.53 |
| 12,13-Epoxy-9-Octadecenoic Acid | Lipids | 25619 | 8961 | 1.08 | -1.52 |
| LysoPC 19:1 | Lipids | 28646 | 10066 | 1.62 | -1.51 |
| LysoPC 18:3(2n isomer) | Lipids | 138417 | 49237 | 1.43 | -1.49 |
| 9(10)-EpOME;(9R,10S)-(12Z)-9,10-Epoxyoctadecenoic acid | Lipids | 45611 | 16297 | 1.16 | -1.48 |
| LysoPC 20:2(2n isomer) | Lipids | 28400 | 10150 | 1.25 | -1.48 |
| 2-Hydroxycinnamic acid | Organic Acids | 137335 | 49293 | 1.21 | -1.48 |
| Azelaic acid | Organic Acids | 1947933 | 702667 | 1.49 | -1.47 |
| LysoPC 20:1 | Lipids | 37200 | 13762 | 1.37 | -1.43 |
| 1-Eicosanol | Lipids | 17845 | 6637 | 1.66 | -1.43 |
| 12-Oxo-phytodienoic acid | Lipids | 6361 | 2366 | 1.35 | -1.43 |
| LysoPC 16:2(2n isomer) | Lipids | 18376 | 6862 | 1.16 | -1.42 |
| Ricinoleic acid | Lipids | 17116 | 6408 | 1.50 | -1.42 |
| Oxiglutatione | Amino Acids and Derivatives | 342517 | 130915 | 1.58 | -1.39 |
| Isoguanine | Nucleotides and Derivatives | 60779 | 23535 | 1.65 | -1.37 |
| Vanillic acid | Phenolic Acids | 33972 | 13323 | 1.35 | -1.35 |
| Caffeic acid | Phenolic Acids | 2695933 | 1059830 | 1.33 | -1.35 |
| N-Feruloyl-3-methoxytyramine | Alkaloids | 19686667 | 7746367 | 1.35 | -1.35 |
| Isoscopoletin (6-Hydroxy-7-Methoxycoumarin) | Lignans and Coumarins | 5870767 | 2314480 | 1.16 | -1.34 |
| β-Nicotyrine | Alkaloids | 309473 | 123500 | 1.67 | -1.33 |
| Isorhamnetin-3-O-rutinoside-4'-O-glucoside | Flavonoids | 7392 | 2970 | 1.15 | -1.32 |
| 9-Hydroxy-12-oxo-15(Z)-octadecenoic acid | Lipids | 15028 | 6050 | 1.19 | -1.31 |
| LysoPC 20:3 | Lipids | 17039 | 6870 | 1.31 | -1.31 |
| Choline Alfoscerate | Lipids | 46087 | 18618 | 1.29 | -1.31 |
| Hypoxanthine | Nucleotides and Derivatives | 56140 | 22871 | 1.25 | -1.30 |
| Cytosine | Nucleotides and Derivatives | 118690 | 48709 | 1.36 | -1.28 |
| 5,7,4'-Trihydroxy-6,8-dimethoxyisoflavone-7-O-galactoside | Flavonoids | 8153 | 3349 | 1.19 | -1.28 |
| Guanosine 5'-monophosphate | Nucleotides and Derivatives | 58341 | 24211 | 1.52 | -1.27 |
| 1-Linolenoyl-rac-glycerol-diglucoside | Lipids | 136831 | 57143 | 1.19 | -1.26 |
| Tamarixetin-3-O-rutinoside | Flavonoids | 27620 | 11797 | 1.17 | -1.23 |
| L-Leucyl-L-phenylalanine | Amino Acids and Derivatives | 37382 | 16060 | 1.28 | -1.22 |
| LysoPC 17:2 | Lipids | 96674 | 41886 | 1.25 | -1.21 |
| 3,4-Dihydroxyacetophenone | Phenolic Acids | 28379 | 12515 | 1.25 | -1.18 |
| Sinapinaldehyde | Phenolic Acids | 1233400 | 551136 | 1.05 | -1.16 |
| Epipinoresinol | Lignans and Coumarins | 21010 | 9653 | 1.19 | -1.12 |
| Piceatannol-3'-O-glucoside | Others | 54876 | 25553 | 1.22 | -1.10 |
| Protocatechuic acid | Phenolic Acids | 51689 | 24185 | 1.26 | -1.10 |
| Capsianoside IV | Terpenoids | 52849 | 24770 | 1.44 | -1.09 |
| Tetrahydroxy-stilbene-O-glucoside | Others | 48906 | 23129 | 1.60 | -1.08 |
| Istanbulin B | Terpenoids | 2411 | 1141 | 1.49 | -1.08 |
| N-Oleoylethanolamine | Alkaloids | 23431 | 11241 | 1.48 | -1.06 |
| Isofraxetin | Lignans and Coumarins | 437927 | 211887 | 1.38 | -1.05 |
| D-Panthenol | Others | 1994 | 966 | 1.11 | -1.05 |
| Thymine | Nucleotides and Derivatives | 4708 | 2291 | 1.46 | -1.04 |
| N-Feruloyltyramine | Alkaloids | 880187 | 429193 | 1.58 | -1.04 |
| Sexangularetin-3-O-glucoside-7-O-rhamnoside | Flavonoids | 147122 | 71785 | 1.07 | -1.04 |
| Hyperin | Flavonoids | 14955 | 7326 | 1.38 | -1.03 |

**Table S8 Differential metabolites upregulated in the PKSe vs PZSe group**

| Compounds | Primary Classification | PKSe | PZSe | VIP | log2FC |
| --- | --- | --- | --- | --- | --- |
| N-Acetyl-L-leucine | Amino Acids and Derivatives | 9 | 4713 | 1.73 | 9.03 |
| 5-O-Feruloylquinic acid | Phenolic Acids | 2294 | 56369 | 1.52 | 4.62 |
| Abscisic acid | Organic Acids | 9931 | 66597 | 1.61 | 2.75 |
| Myosmine | Alkaloids | 10079 | 58304 | 1.72 | 2.53 |
| Scopoletin-7-O-glucuronide | Lignans and Coumarins | 380873 | 1916570 | 1.52 | 2.33 |
| Chlorogenic acid methyl ester | Phenolic Acids | 663900 | 3234900 | 1.50 | 2.28 |
| 1-O-Feruloylquinic acid | Phenolic Acids | 394207 | 1783297 | 1.50 | 2.18 |
| Asperulosidic acid | Terpenoids | 5146 | 19753 | 1.67 | 1.94 |
| L-Lysine-Butanoic Acid | Amino Acids and Derivatives | 2092 | 8007 | 1.38 | 1.94 |
| Dimethylmalonic acid | Organic Acids | 16062 | 59925 | 1.23 | 1.90 |
| 2-Methylsuccinic acid | Organic Acids | 145970 | 484657 | 1.34 | 1.73 |
| 5'-Methoxyisolariciresinol-9'-O-glucoside | Lignans and Coumarins | 18321 | 58683 | 1.46 | 1.68 |
| 2-Hydroxy-2-methyl-3-oxobutanoic acid | Organic Acids | 14760 | 46523 | 1.27 | 1.66 |
| 5-Oxo-L-Proline | Amino Acids and Derivatives | 6577 | 20541 | 1.59 | 1.64 |
| 3-Hydroxy-3-methylpentane-1,5-dioic acid | Amino Acids and Derivatives | 875733 | 2644167 | 1.64 | 1.59 |
| Caffeine | Alkaloids | 4421 | 13143 | 1.12 | 1.57 |
| Pipecolic acid | Amino Acids and Derivatives | 1007793 | 2238167 | 1.45 | 1.15 |
| Dihydromyricetin-3-O-glucoside | Flavonoids | 10401 | 22950 | 1.41 | 1.14 |
| 5-Acetamidopentanoic Acid | Organic Acids | 175890 | 364317 | 1.39 | 1.05 |
| L-Citrulline | Amino Acids and Derivatives | 83651 | 170007 | 1.45 | 1.02 |

**Table S9 DEGs associated with the KEGG metabolic pathway**

| Gene symbol | Gene ID | log2FC | pvalue | Annotation | pathway_id | pathway_name |
| --- | --- | --- | --- | --- | --- | --- |
| CCR-1 | gene-LOC107765220 | -10.78 | 2.83E-16 | cinnamoyl-CoA reductase | ko00940 | Phenylpropanoid biosynthesis |
| CCR-2 | gene-LOC107765221 | -11.79 | 1.81E-38 |  |  |  |
| CCR-3 | gene-LOC107795353 | -1.11 | 3.15E-05 |  |  |  |
| APRT | gene-LOC107787030 | -4.775 | 1.04E-03 | adenine phosphoribosyltransferase | ko00230 | Purine metabolism |
| XDH-1 | gene-LOC107800936 | -1.354 | 7.27E-03 | Xanthine Dehydrogenase |  |  |
| XDH-2 | gene-LOC107799132 | -2.341 | 1.24E-08 |  |  |  |
| amn | gene-LOC107819006 | -10.633 | 6.51E-16 | AMP nucleosidase |  |  |

**Table S10 DEGs associated with the KEGG metabolic pathway**

| Gene symbol | Gene ID | log2FC | pvalue | Annotation | pathway_id | pathway_name |
| --- | --- | --- | --- | --- | --- | --- |
| AK | gene-LOC107773218 | -1.123 | 3.32E-03 | adenylate kinase | ko00230 | Purine metabolism |
| CCR-1 | gene-LOC107767921 | 2.38 | 1.93E-07 | cinnamoyl-CoA reductase | ko00940 | Phenylpropanoid biosynthesis |
| CCR-2 | gene-LOC107796275 | 3.49 | 2.55E-05 |  |  |  |
| CCR-3 | gene-LOC107816628 | 1.24 | 1.40E-03 |  |  |  |
| CCR-4 | gene-LOC107765220 | -11.085 | 1.27E-19 |  |  |  |
| CCR-5 | gene-LOC107765221 | -13.525 | 5.30E-25 |  |  |  |
| C3'H-1 | gene-LOC107792896 | -6.409 | 6.15E-04 | 5-O-(4-coumaroyl)-D-quinate 3'-monooxygenase |  |  |
| C3'H-2 | gene-LOC107800961 | -4.375 | 1.13E-04 |  |  |  |
| 4CL | gene-LOC107803673 | 1.062 | 2.76E-03 | 4-coumarate--CoA ligase |  |  |
| CAD-1 | gene-LOC107763167 | 2.935 | 1.28E-03 | cinnamyl-alcohol dehydrogenase |  |  |
| CAD-2 | gene-LOC107789497 | 3.681 | 5.74E-05 |  |  |  |
| CAD-3 | gene-LOC107797120 | 2.543 | 2.82E-04 |  |  |  |
| CAD-4 | gene-LOC107798749 | 2.79 | 3.16E-03 |  |  |  |


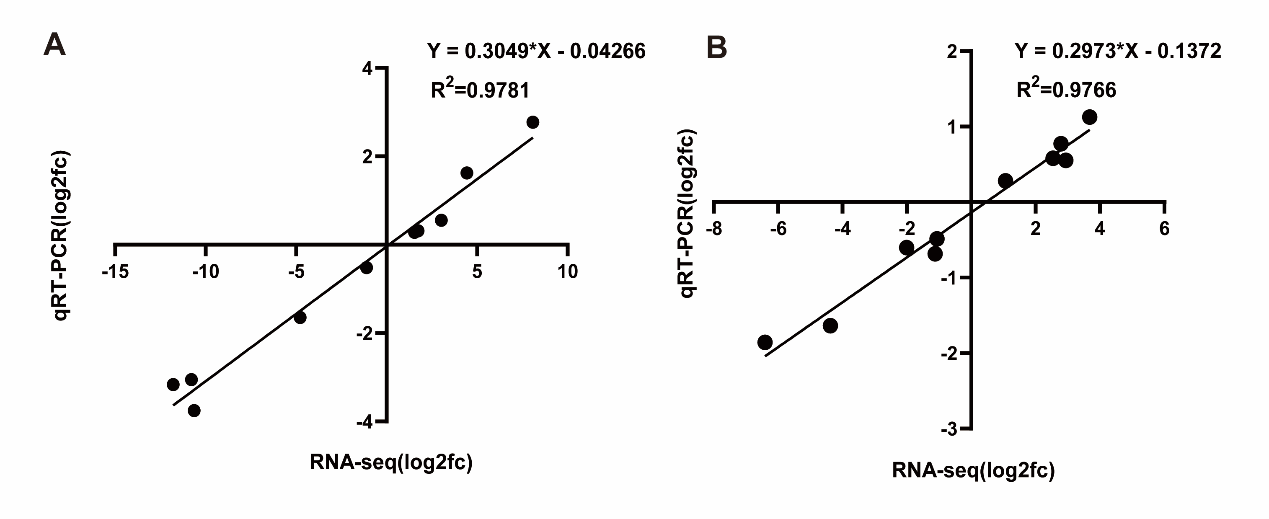


Fig. S1 qPCR validation of differentially expressed genes in metabolic pathways. A is PK vs PZ Group, B is PKSe vs PZSe group.
